# Sertoli Cell‐Derived Extracellular Vesicles Orchestrate Cadmium‐Induced Testicular Inflammation and Fibrosis

**DOI:** 10.1002/advs.202522278

**Published:** 2026-03-25

**Authors:** Jianfeng Ma, Mailin Gan, Shuang Liang, Siyu Chen, Ziling Hao, Yiting Yang, Jiawei Lu, Qihang Wu, Yuqian Shi, Lijun Sun, Jiaxin Li, Saihao Wang, Yan Wang, Xiaofeng Zhou, Lei Chen, Ye Zhao, Li Zhu, Linyuan Shen

**Affiliations:** ^1^ Farm Animal Germplasm Resources and Biotech Breeding Key Laboratory of Sichuan Province Sichuan Agricultural University Chengdu China; ^2^ State Key Laboratory of Swine and Poultry Breeding Industry Sichuan Agricultural University Chengdu China

**Keywords:** cadmium, extracellular vesicles, testicular fibrosis, programmed cell death

## Abstract

Cadmium (Cd) is a widespread environmental toxicant that impairs male reproductive health, though its testicular toxicity mechanisms remain incompletely defined. Combining single‐cell RNA sequencing with functional assays, we identified a novel intercellular communication pathway mediated by Sertoli cell‐derived extracellular vesicles (EVs) in Cd‐induced testicular injury. In mice, Cd exposure caused testicular atrophy, spermatogenesis disruption, and fibrosis. Multi‐omics analyses revealed activation of multiple programmed cell death pathways, including apoptosis, necroptosis, pyroptosis, and ferroptosis. Single‐cell RNA sequencing (scRNA‐seq) demonstrated testicular cellular remodeling featuring Sertoli cell depletion and fibroblast expansion. Mechanistically, Cd triggered multi‐modal programmed cell death (PCD) in Sertoli cells, promoting EV release enriched with damage‐associated molecular patterns (DAMPs) and mitochondrial components. These EVs were internalized by testicular macrophages, activating the TLR4/NF‐κB pathway and inducing a pro‐inflammatory phenotype. Consequently, activated macrophages stimulated fibroblast‐mediated fibrosis via TGF‐β/Smad signaling. These findings elucidate a Sertoli cell‐EV‐macrophage‐fibroblast axis in Cd‐induced testicular damage, offering new insights into environmental toxicant‐induced male infertility and potential therapeutic targets.

## Graphical abstract

Environmental cadmium exposure disrupts testicular homeostasis through a novel intercellular communication axis. Stressed Sertoli cells release extracellular vesicles carrying damage‐associated molecular patterns and mitochondrial fragments, which activate macrophages via TLR4/NF‐κB signaling. These inflammatory macrophages subsequently stimulate fibroblast activation and collagen deposition through TGF‐β/Smad pathway, leading to testicular fibrosis. These findings reveal potential therapeutic targets for environmental toxicant‐induced male infertility.

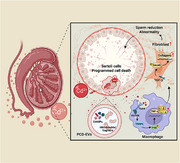

## 1 Introduction

Cadmium (Cd) is a pervasive environmental contaminant and occupational hazard, released into the environment through industrial emissions, agricultural activities, and tobacco smoke [1]. Its persistence results in widespread accumulation in water and food sources, posing a significant risk to human health [2]. However, because of its long biological half‐life and slow metabolism, Cd readily accumulates in human tissues, especially reproductive system, posing a significant public health risk [3]. Epidemiological and meta‐analytic studies demonstrate a strong link between Cd exposure and male infertility. These elevated levels in blood, semen, or urine are consistently correlated with impaired sperm quality, aberrant semen parameters, and disrupted sex hormone levels [4]. Therefore, it is urgent to take measures to intervene in the impact of Cd exposure on male infertility.

Extensive research has established that Cd exerts pronounced testicular toxicity. Cd exposure leads to testicular atrophy, severe disruption of seminiferous tubule architecture, ultimately impairing spermatogenesis and reducing testicular weight [5, 6]. At the cellular level, Cd induces oxidative stress, disrupts the blood‐testis barrier (BTB), and triggers apoptosis in both germ cells and somatic cells, including Sertoli and Leydig cells [7]. Mechanistically, the understanding of Cd reproductive toxicity has focused on its direct cytotoxic effects, including the generation of reactive oxygen species (ROS), mitochondrial dysfunction, and activation of cell death pathways [5, 8, 9]. However, there is still a lack of complete understanding of the complex testicular microenvironment after Cd exposure.

Spermatogenesis and testicular homeostasis rely on the coordinated function of diverse cell populations within the testis. In the interstitial space, Leydig cells are responsible for testosterone production and paracrine signaling [10]. Testicular macrophages and endothelial cells further modulate the immune environment and vascularization [11, 12]. Sertoli cells are central players in the maintenance of the blood‐testis barrier (BTB) that provides nutritional and structural support for spermatogenesis [13]. In most studies, the primary focus has been on the direct cytotoxic effects of cadmium on a single cell type. However, the critical role of intercellular communication in orchestrating the overall testicular injury remains largely overlooked. In this context, extracellular vesicles (EVs) have emerged as pivotal mediators of cell‐to‐cell communication, carrying a complex cargo of proteins, lipids, and nucleic acids that can profoundly alter the function of recipient cells [14]. Under physiological conditions, EVs facilitate the transfer of bioactive molecules and regulatory signals between cells, thus orchestrating processes such as germ cell differentiation, immune regulation, and maintenance of testicular homeostasis [12]. Importantly, EVs can traverse the BTB, enabling communication between the seminiferous tubules and interstitial compartments [12, 14]. In pathological states, stressed cells release EVs enriched with damage‐associated molecular patterns (DAMPs) [15]. Such EV‐mediated signaling has been implicated in the propagation of injury and the development of disease in various organs [16]. Despite advances in understanding Cd‐induced testicular toxicity, significant knowledge gaps remain regarding the cell type‐specific stress responses and intercellular communication networks within the testis under Cd exposure. Although EVs are recognized as key mediators of intercellular communication, their specific functions in orchestrating Cd testicular toxicity remain unexplored.

Here, our study used multiple omics analyses to elucidate the mechanism of Cd induced testicular injury, providing a unique perspective on the intercellular communication in testis at the single cell level. Moreover, we investigated the underlying mechanism of extracellular vesicles derived from Sertoli cells mediating testicular fibrosis.

## 2 Results

### 2.1 Testicular Injury and Spermatogenesis Disorder in Cd Exposed Mouse Model

**FIGURE 1:**
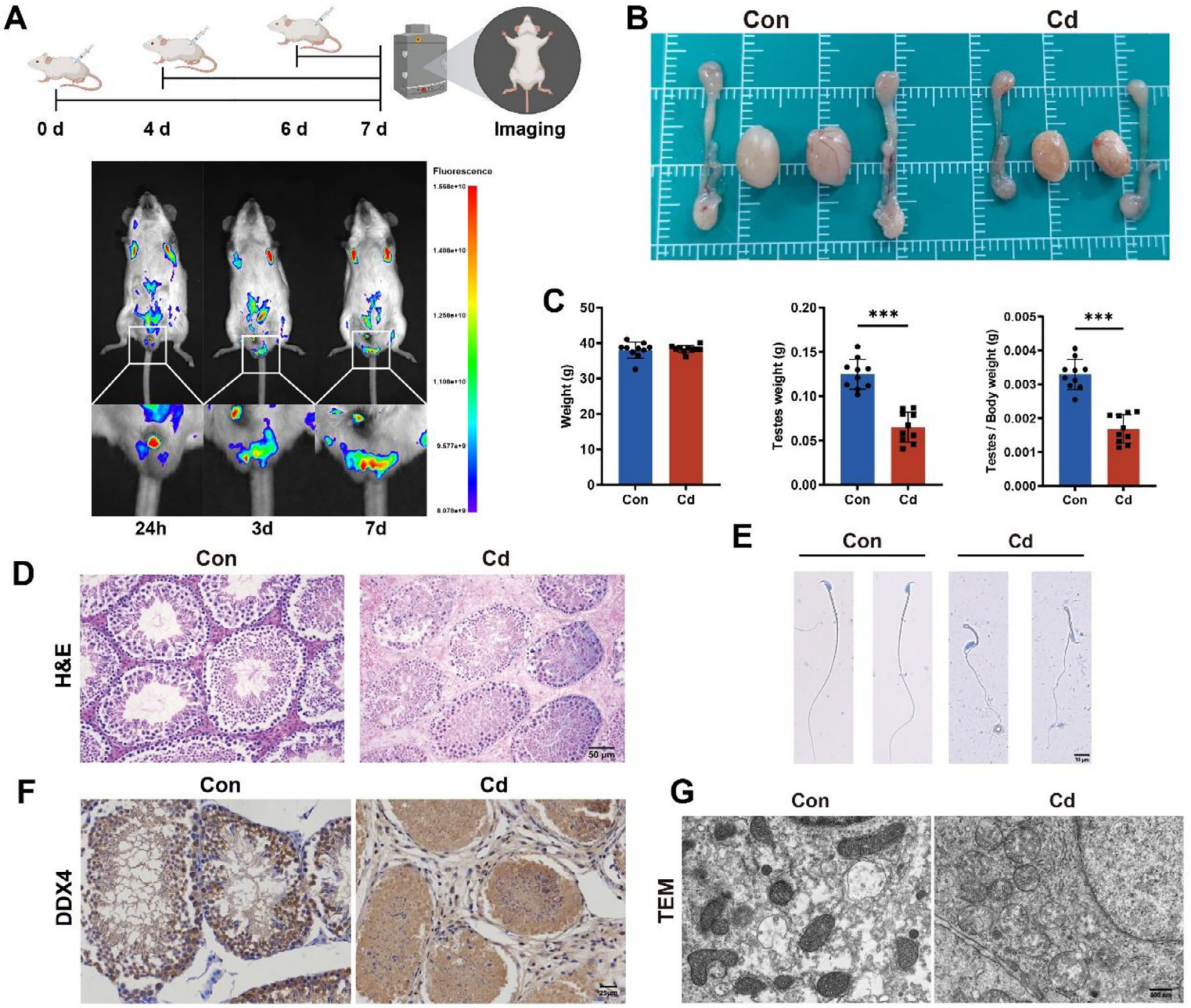
Cadmium exposure induces testicular injury. **(A)** In vitro fluorescence imaging of cadmium probe after cadmium injection 24 h, 3 day, 7 day. **(B)** Image of mice testis in control group (Con) and cadmium treatment group (Cd). **(C)** Body weight, testis weight, and testis/body weight ratios of Con and CD mice. Results were plotted as means ± SEM, ***p < 0.001, n = 10. **(D)** H&E staining of testis tissue sections. **(E)** Representative images of sperm in epididymal sperm of Con and Cd mice. **(F)** Immunohistochemical staining of testis tissues for DDX4. **(G)** Representative transmission electron microscope (TEM) images of testis tissue.

We analyzed the relative levels of Cd in mouse testis at 24 h, 3 days, and 7 days after acute Cd exposure using cadmium probes. The results of live‐imaging showed a progressive increase of the fluorescent signal within testis indicates Cd accumulation (Figure 1A). At the 8 day post‐exposure, Cd exposure caused no change in body weight compared with the control group, but led to a decrease in testis weight (Figure 1C). There was obvious adhesion tissue in the scrotum. The testis was seriously damage and tissue texture became hard (Figure 1B). H&E stained section showed the seminiferous tubules were atrophic, and interstitial space widened and was filled with red matrix in Cd group (Figure 1D). The epididymal sperm were rare and showed abnormal bending in Cd group (Figure 1E). Cd exposure led to a decrease in DDX4 expression, a pan‐germ cell marker (Figure 1F). Transmission electron microscope (TEM) imaging identified swollen mitochondria characterized by disrupted membranes in Cd group testis (Figure 1G). Additionally, testosterone levels in the plasma of mice were reduced following cadmium exposure, while FSH and LH showed no significant changes (Figure S1).

### 2.2 Transcriptome Reveals Programmed Cell Death in Cd‐Exposed Testis

**FIGURE 2:**
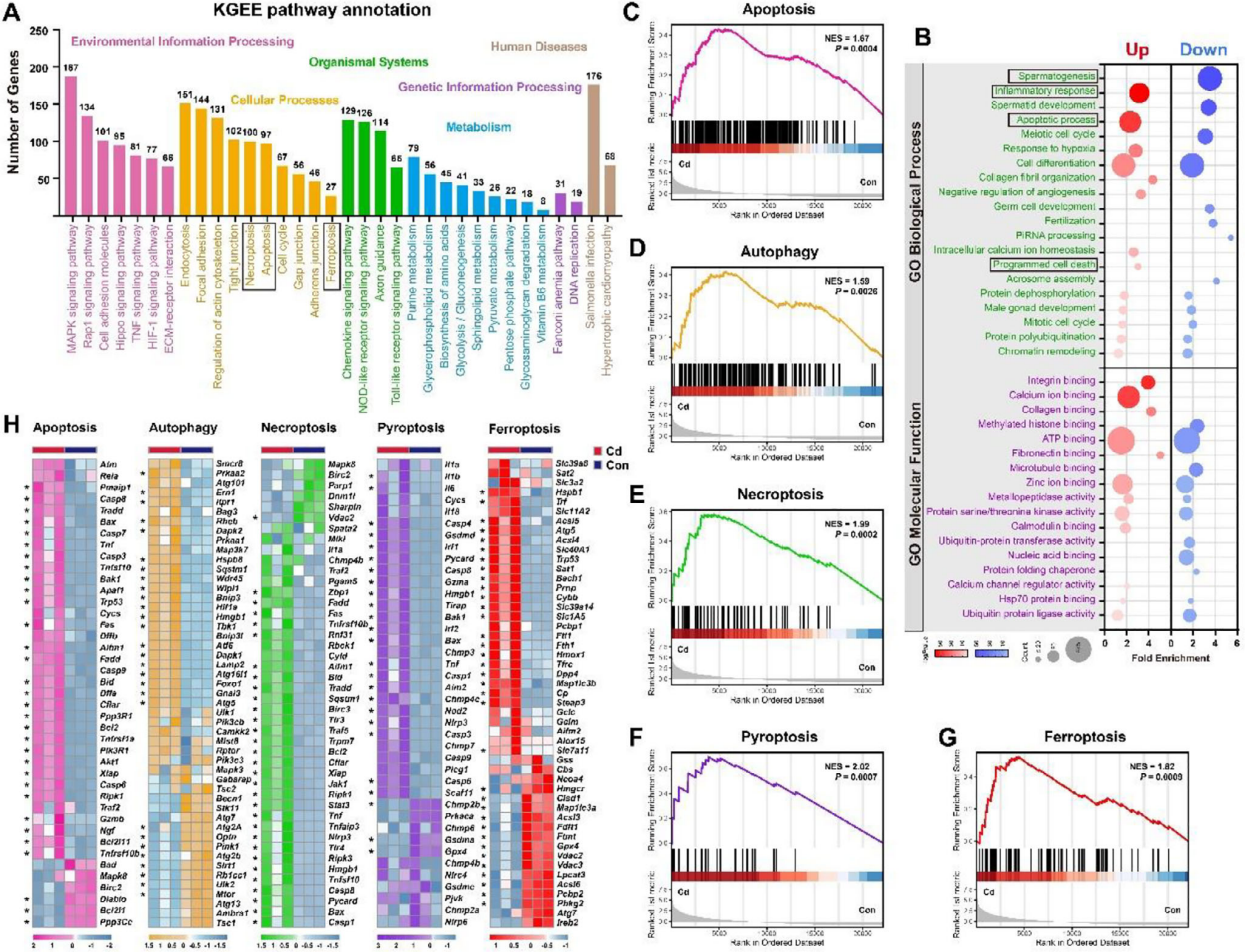
Transcriptomic analysis of cadmium‐induced testicular injury. **(A)** KEGG pathway annotation of differentially expressed genes (DEGs). **(B)** Gene ontology (GO) molecular function and biological process enrichment analysis of differentially expressed genes. Gene set enrichment analysis of apoptosis **(C)**, autophagy **(D)**, necroptosis **(E)**, pyroptosis **(F)**, and ferroptosis **(G)** related genes. **(H)** Heatmap of programmed cell‐death related genes expression.

High‐throughput transcriptome analysis of the testis in the Cd and control groups was performed. Transcriptome analysis identified 5993 upregulated genes and 7530 downregulated genes (Figure S2A,B). The KEGG pathway annotation of differentially expressed genes (DEGs) involves cellular processes including cell cycle, cell necroptosis, apoptosis, and ferroptosis. They also involved the TNF signaling pathway and Toll‐like receptor signaling pathway (Figure 2A). GO enrichment analysis indicated that DEGs were enriched into spermatogenesis, inflammatory response, programmed cell death (Figure 2B). To further explore the cell death pathway, we conducted the gene set enrichment analysis (GSEA) on various programmed cell death. GSEA results revealed that the apoptosis, autophagy, necroptosis, pyroptosis, and ferroptosis were significantly enriched, and cuproptosis was not significant (Figure 2C‐G; Figure S2C). The heatmap showed the expression of various programmed cell death pathways‐related genes. Most of the related genes were upregulated in Cd group (Figure 2H). We further investigated testicular proteome (Figure S3A,B), and classified differentially expressed proteins (DEPs) using PANTHER Classification System (Figure S3C). The transcriptome and proteome data were performed with a global correlation analysis to identify up‐regulated and down‐regulated DEPs (Figure S3D,E). GO and KEGG functional enrichment analyses also revealed DEPs were involved in apoptosis, ferroptosis, extracellular martrix structual constituent, TNF signaling pathway (Figure S3F–H).

### 2.3 Single‐Cell RNA Sequencing (scRNA‐seq) Reveals Cell Population Characterization in Cadmium Exposed‐Testis

**FIGURE 3:**
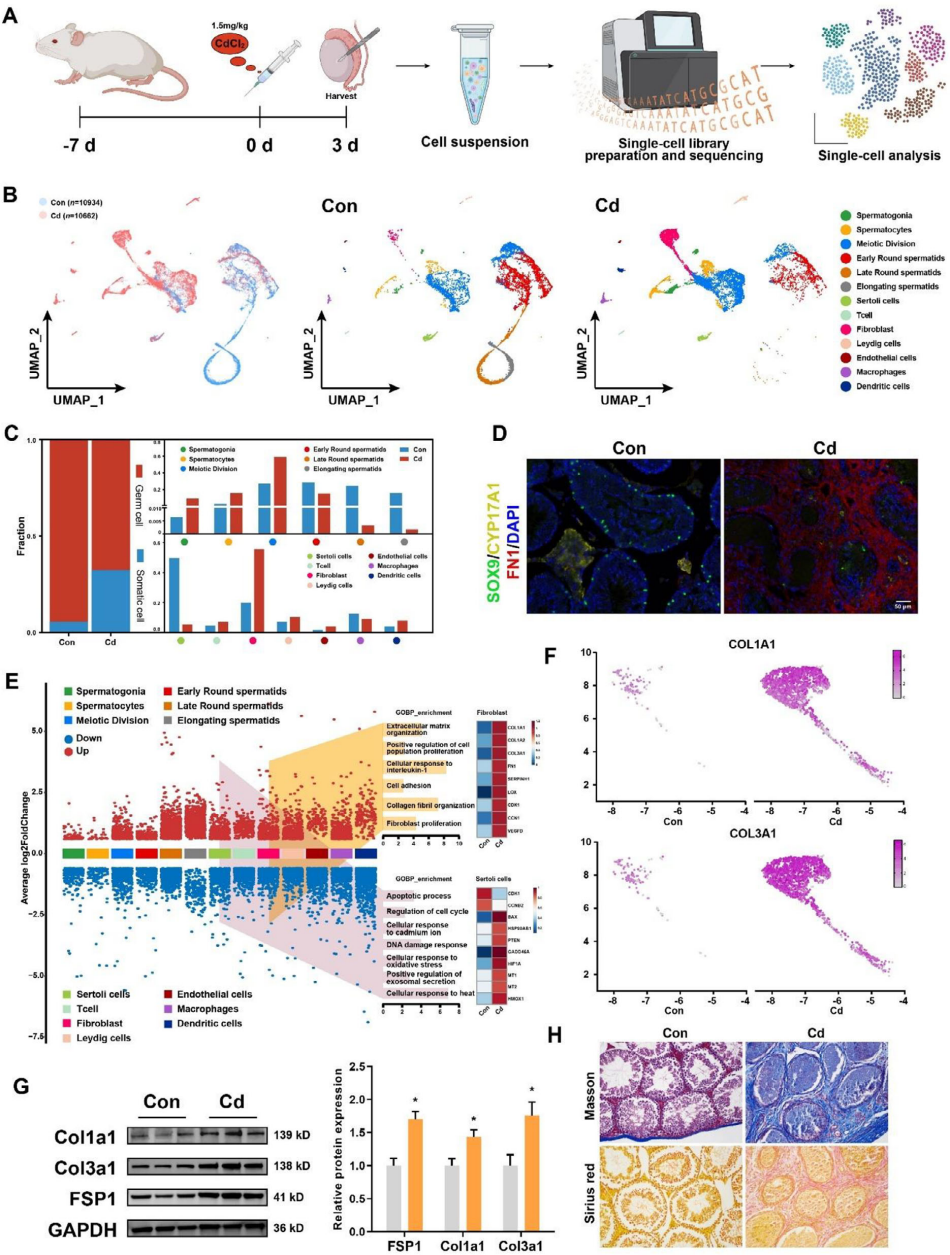
Single‐cell RNA sequencing (scRNA‐seq) analysis of Cd‐induced testicular injury. **(A)** The experimental workflow of the isolation of cells from testis tissues for scRNA‐seq. **(B)** UMAP plot for integrating two group of single‐cell sequencing data (left panel) and showing cell types from control (middle panel) and Cd‐exposed (right panel) group testis. **(C)** The composition ratio of major cell types in control and Cd‐exposed testis. **(D)** Immunofluorescence images of Sertoli cell marker SOX9, leydig cell marker CYP17A1, and fibroblast marker FN1. **(E)** Differentially expressed genes (DEGs) in various cell types between the control group and the Cd‐exposed group. Right panel shows the Gene Ontology enrichment analysis and heatmap of related gene expression for DEGs in Sertoli cells and fibroblasts. **(F)** UMAP plots showing expression of COL1A1 and COL3A1 in fibroblasts. **(G)** Western blot analysis and quantification for Col1a1, Col3a1, and FSP1 in control and Cd‐exposed testis. **(H)** Masson and Sirius red staining of testis sections. Results represent mean ± SEM, *p < 0.05, n = 3.

To investigate the changes in cell population in the Cd‐exposed testis, we performed scRNA‐seq on mice testis tissue at three days after Cd exposure (Figure 3A). A total of 21 596 cells (10 934 for control group and 10 662 for Cd exposure group) were retained for further analysis, after data quality control at the gene and cell levels (Figure 3B). We identified 13 major cell types (Figure 3B) according to the expression of canonical markers in mouse testis (Figure S4), including Spermatogonia, Spermatocytes, Meiotic Division cell, Early round spermatids, Late round spermatids, Sertoli cells, T cell, Fibroblast, Leydig cells, Endothelial cells, Macrophages, Dendritic cells. We further calculated the proportion of different types of cells. The proportion of germ cells in the testis of Cd exposed mice decreased, while the proportion of somatic cells increased (Figure 3C). Moreover, the proportion of fibroblasts was sharply increased, while the proportion of Sertoli cells decreased (Figure 3C). Immunofluorescence analysis of testis tissue confirmed decreased expression of the Sertoli cells marker SOX9 and increased the expression of the fibroblast marker FN1 (Figure 3D). We then conducted differentially expressed gene (DEGs) and functional enrichment analysis on the annotated cell types. The DEGs in testicular fibroblasts after Cd exposure involved in fibroblast proliferation, collagen fibril organization, cellular response to interleukin‐1 (Figure 3E). The DEGs in Sertoli cells mainly involved in apoptotic process, regulation of cell cycle, DNA damage response (Figure 3E). UMAP plots show the expression of COL1A1 and COL3A1 (collagen fibers marker) in fibroblasts were significantly increased in Cd group (Figure 3F). Western blot result also indicated that Col1a1, Col3a1, and FSP1 expression level increased in Cd‐exposed testis (Figure 3G). This increased collagen fibers were further supported with the comparison of Masson staining and Sirius red staining (Figure 3H).

### 2.4 Cadmium Induces Sertoli Cells Programmed Cell Death

**FIGURE 4:**
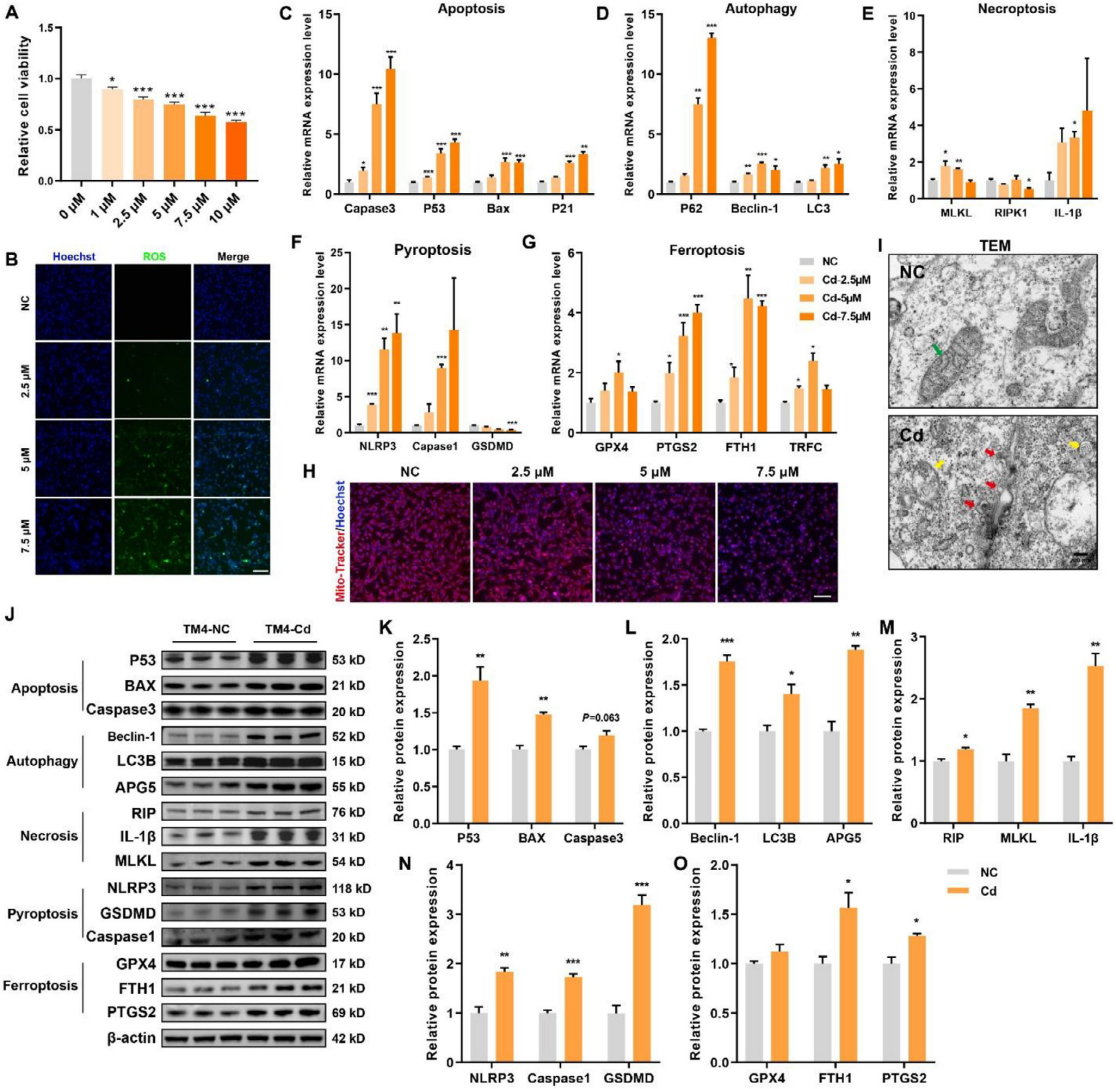
Cadmium induces Sertoli cells programmed cell death (PCD) in vitro. **(A)** Cell viability by CCK‐8 assay. **(B)** ROS contents estimated by ROS assay. Scale bar  =  100 µm. RT‐qPCR analysis of apoptosis **(C)**, autophagy **(D)**, necroptosis **(E)**, pyroptosis **(F)**, and ferroptosis **(G)** PCD‐related genes mRNA expression. **(H)** Detection of mitochondrial content in cadmium treated Sertoli cells using Mitotracker. Scale bar  =  100 µm. **(I)** Representative transmission electron microscope (TEM) images of Sertoli cells. Green arrow, normal mitochondria; Yellow arrow, damaged mitochondria; Red arrow, secretory vesicles. **(J)** Expression levels of PCD‐related proteins by western blot analysis in cadmium‐treated (5 µm) Sertoli cells. **(K‐O)** Quantification of Western blot results. Results represent mean ± SEM, ***p < 0.001, **p < 0.01, *p < 0.05, n = 3.

We constructed the Cd‐exposed Sertoli cell model in vitro (Figure 4A). Cd exposure led to significant increase in intracellular reactive oxygen species (ROS) (Figure 4B). The expression of the programmed cell death hallmark genes showed an upward trend in Cd‐exposed Sertoli cells, including apoptosis, autophagy, necroptosis, pyroptosis, and ferroptosis (Figure 4C–G). MitoTracker staining showed a lower mitochondrial membrane potential in Cd‐exposed Sertoli cells (Figure 4H). Transmission electron microscopy revealed disruptions in mitochondrial structure in Cd‐exposed Sertoli cells (Figure 4I). Western blot analysis also confirmed that Cd exposure significantly increases apoptosis, autophagy, necrosis, pyroptosis, and ferroptosis‐related protein expression in Sertoli cells (Figure 4J–O).

### 2.5 PCD‐Sertoli Cells Release Extracellular Vesicles Carrying DAMPs

**FIGURE 5:**
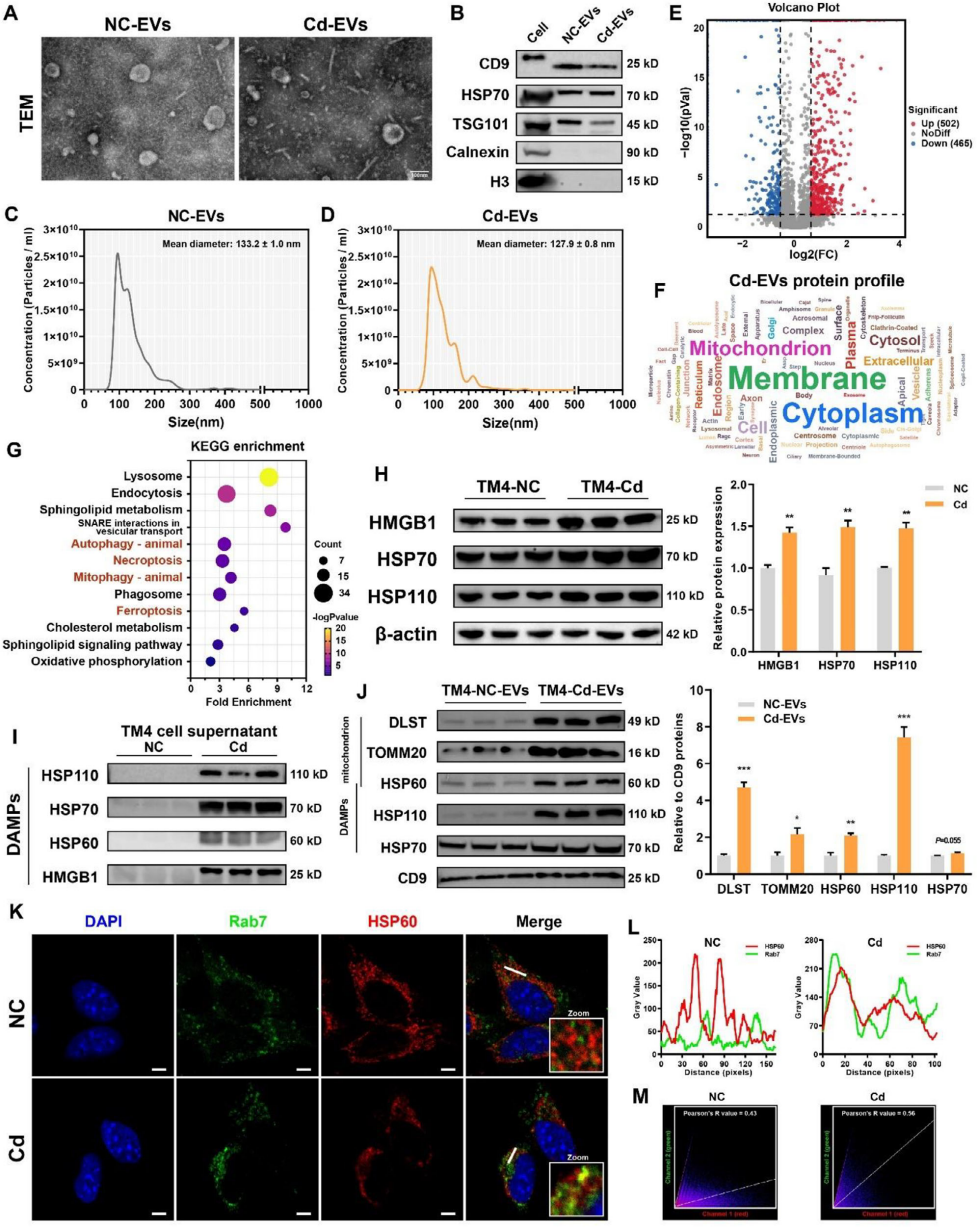
PCD‐Sertoli cells release extracellular vesicles (EVs) carrying damage‐associated molecular pattern molecules (DAMPs). **(A)** Transmission electron microscopy (TEM) image of extracellular vesicle for Sertoli cells from negative control group (NC‐EVs) and Cd treatment group (Cd‐EVs). **(B)** Western blot analysis of the EVs surface biomarkers CD9, HSP70, TSG101, and Calnexin. **(C‐D)** Nanoparticle tracking analysis (NTA) of NC‐EVs and Cd‐EVs. **(E)** Volcano plot of differentially expressed proteins between Cd‐EVs and NC‐EVs quantified by proteomic analysis. **(F)** Word cloud image illustrating the subcellular localization annotations found in the increased protein in Cd‐EVs. **(G)** KEGG enrichment of differentially expressed proteins between Cd‐EVs and NC‐EVs. **(H)** Western blot analysis of DAMPs level in negative control (NC) and Cd treatment (Cd) Sertoli cells. **(I)** Western blot analysis of DAMPs level in the Sertoli cells culture supernatant. **(J)** Western blot analysis of DAMPs and mitochondrial protein DLST, TOMM20, and HSP60 in Sertoli cells EVs. **(K)** The distribution and colocalization of late endosomes proteins Rab7 and mitochondrial protein HSP60 in NC and Cd Sertoli cells. Nuclei were stained with DAPI (blue). Scale bar = 5 µm. **(L)** Gray‐value quantitative colocalization analysis in selected areas using ImageJ software. **(M)** Rab7 and HSP60 signal overlap of colocalization was analyzed with Pearson's correlation using ImageJ. Results represent mean ± SEM, ***p < 0.001, **p < 0.01, *p < 0.05, n = 3.

In scRNA‐seq data analysis, we noticed that differentially expressed genes in Sertoli cells were enriched in the regulation of exosomal secretion (Figure 3E; Figure S4C). Extracellular vesicles (EVs) from Sertoli cells were extracted from the cell culture supernatant using ultracentrifugation, and TEM imaging was performed to show EVs morphology (Figure 5A). Western blot further showed the isolated EVs expressed the markers of CD9, HSP70, and TSG101 without the cell‐specific marker Calnexin and H3 (Figure 5B). The mean diameter of EVs derived from Sertoli cells in the negative control (NC‐EVs) and Cd‐exposed group (Cd‐EVs) was 133.2 ± 1.0 nm and 127.9 ± 0.8 nm (Figure 5C,D). To evaluate the contents of the EVs, qualitative proteomic analysis was performed. A total of 502 up‐regulated and 465 down‐regulated proteins were identified in Cd‐EVs (Figure 5E). Subcellular localization annotations of up‐regulated proteins were classified. The main categories included the cytoplasm, mitochondrion, membrane (Figure 5F). KEGG enrichment analysis indicated that up‐regulated proteins were mainly enriched in necroptosis, mitophagy, and ferroptosis (Figure 5G). Furthermore, we found that the damage‐associated molecular pattern (DAMPs) HMGB1, HSP70, and HSP110 were increased in Cd‐exposed Sertoli cells and the cell culture supernatant (Figure 5H,I). DAMPs levels were also found to be significantly increased in Cd‐EVs (Figure 5J). We further detected the mitochondrial protein level in the EVs. Protein levels of DLST, TOMM20, and HSP60 in the Cd‐EVs were significantly increased compared with those in the NC‐EVs (Figure 5J). Immunofluorescence assay demonstrated colocalization between the mitochondrial marker HSP60 and the late endosome marker Rab7 (Figure 5K). Increased correlations were observed between HSP60 and Rab7 in Cd‐exposed Sertoli cells (Figure 5L,M). These results indicate that Cd exposure triggers the secretion of DAMP molecules and mitochondrial fragments into the extracellular space via EVs by inducing programmed death in Sertoli cells.

### 2.6 Extracellular Vesicles Released From PCD‐Sertoli Cells Activate Macrophages and Induce Inflammation

**FIGURE 6:**
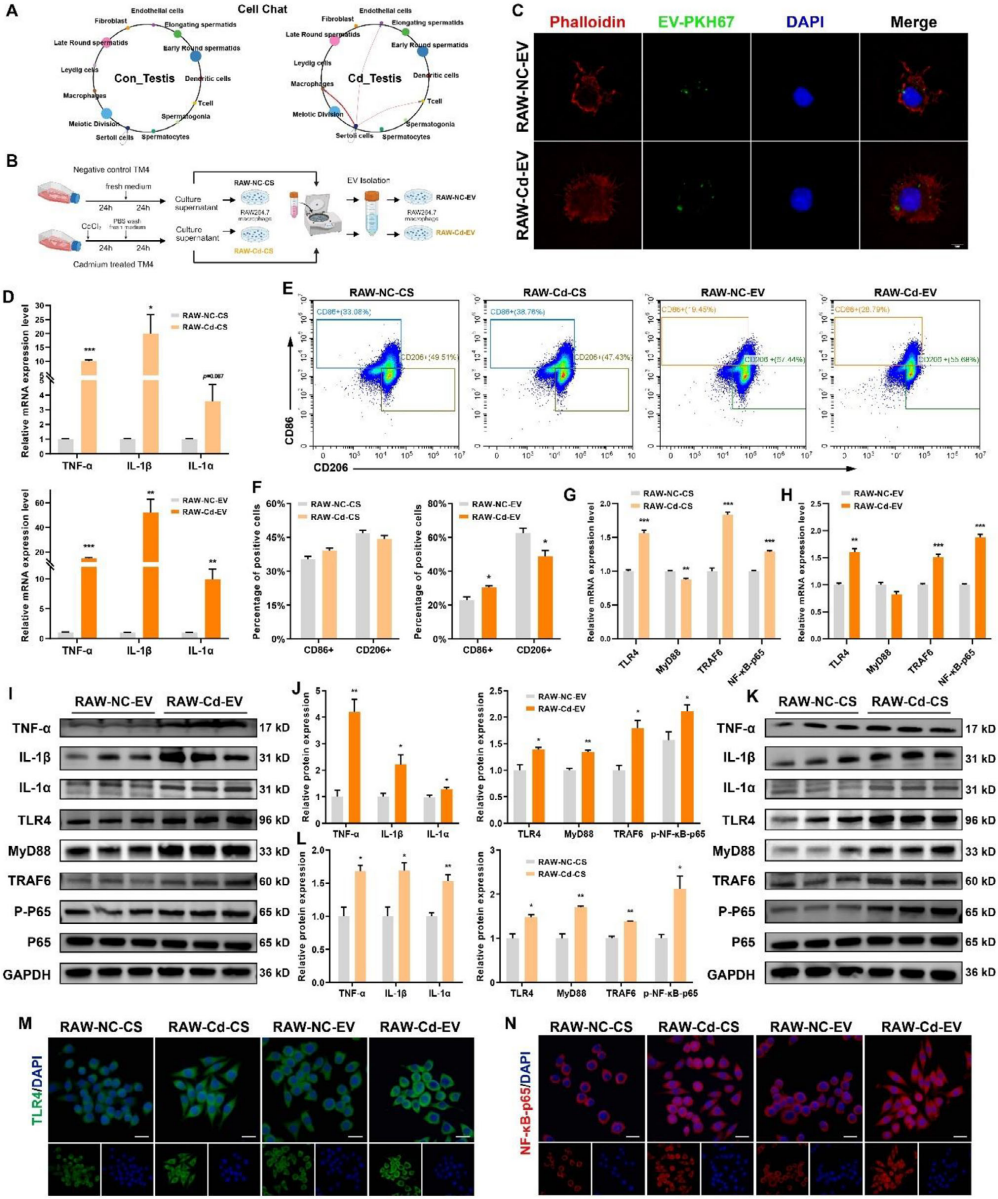
Extracellular vesicles released by PCD‐Sertoli cells activate macrophages and cause inflammation. **(A)** The weight of interactions between different cell types as identified by CellChat analysis. **(B)** Schematic representation of experimental design. **(C)** Sertoli cells‐derived extracellular vesicles (EVs) be taken up by macrophages (RAW). EVs were labeled by PKH67 (green), and cytoskeleton of RAW 264.7 cells was stained by phalloidin (red). **(D)** TNF‐α, IL‐1β, and IL‐1α mRNA expressions from RAW 264.7 cells treated with culture supernatant or EVs of negative control and Cd‐treated Sertoli cells were analyzed by RT‐qPCR. **(E)** Flow cytometry analysis of percentages of CD86^+^ and CD206^+^ cells. **(F)** Statistical analysis of the percentages of CD86^+^ and CD206^+^ macrophages. **(G‐H)** TLR4, MyD88, TRAF6, p65 mRNA expression were tested by RT‐qPCR. **(I‐J)** Western blot of inflammatory factors and TLR4/NF‐κB signaling pathway protein in RAW‐NC‐EV and RAW‐Cd‐EV. **(K‐L)** Western blot of inflammatory factors and TLR4/NF‐κB signaling pathway protein in RAW‐NC‐CS and RAW‐Cd‐CS. Immunofluorescent staining for TLR4 **(M)** and NF‐κB‐p65 **(N)** in each group. Results represent mean ± SEM, ***p < 0.001, **p < 0.01, *p < 0.05, n = 3.

Intercellular communication between different cell types was next inferred using CellChat. Compared with the control group, the total number of interactions increased in Cd‐exposed testis (Figure S5A). A strong interaction signal was detected between Sertoli cells and macrophages after Cd‐exposed (Figure 6A). Immunofluorescence results showed that CD86 signaling was enhanced in Cd exposed testes, and Sertoli cells (SOX9 positive cell) were spatially adjacent to macrophages (Figure S6). The following experiments were designed to validate their potential interaction (Figure 6B). PKH67‐labeled Sertoli cell‐derived EVs were treated with macrophages for 12 h. Phalloidin labeled macrophages could internalize Sertoli cell‐derived EVs labeled with PKH26 (Figure 6C). In macrophages, the mRNA expression level of TNF‐α, IL‐1β, IL‐1α significantly increased after the cells were treated with the cell supernatant (Cd‐CS) or extracellular vesicles (Cd‐EV) derived from Cd‐exposed Sertoli cells (Figure 6D). Flow cytometry demonstrated that Cd‐EV significantly elevated the level of macrophage activation marker CD86 (Figure 6E,F). Cd‐CS or Cd‐EV from Cd‐exposed Sertoli cells upregulated the mRNA expression of TLR4, TRAF6, and NF‐κB‐p65 in macrophages (Figure 6G,H). Similarly, Western blot analysis showed that Cd‐EV and Cd‐CS upregulated the protein expression of TLR4, MyD88, TRAF6, p‐NF‐κB‐p65, and inflammatory factors in macrophages (Figure 6I–L). Immunofluorescence staining revealed that Cd‐CS and Cd‐EV upregulated TLR4 expression (Figure 6M) and induced nuclear translocation of NF‐κB‐p65 (Figure 6N).

### 2.7 Inflammatory Macrophages Activate Fibroblasts and Promote Fibrosis

**FIGURE 7:**
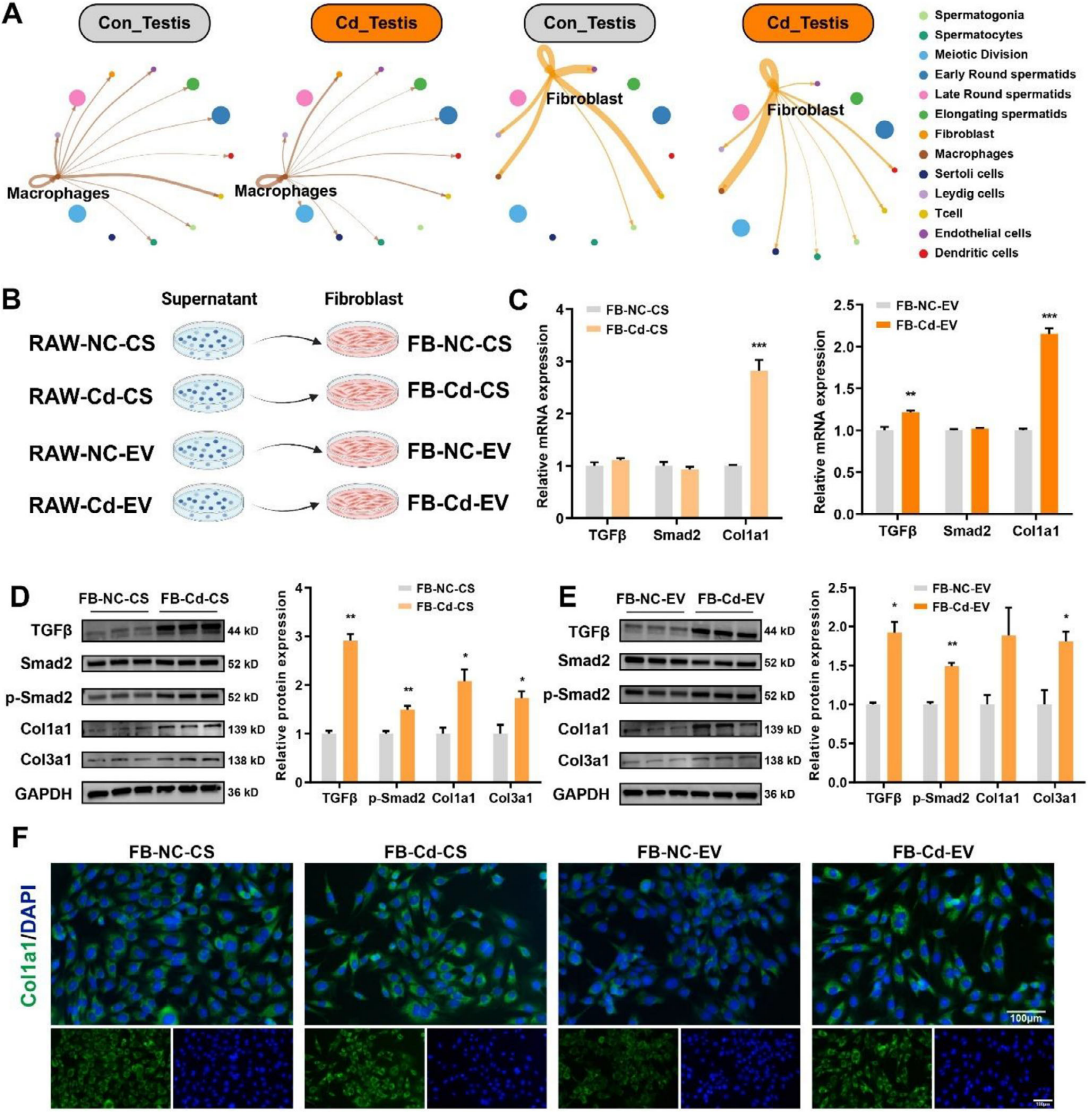
Inflammatory macrophages activate fibroblasts and promoting fibrosis. **(A)** The weight of interactions between macrophages, fibroblasts, and other cells identified by CellChat analysis. **(B)** Schematic representation of experimental design. 3T3‐L1 fibroblasts were treated with the culture supernatant of RAW‐NC‐CS, RAW‐Cd‐CS, RAW‐NC‐EV, RAW‐Cd‐EV groups and named FB‐NC‐CS, FB‐Cd‐CS, FB‐NC‐EV, FB‐Cd‐EV, respectively. **(C)** TGFβ, Smad2, and Col1a1 mRNA expressions from fibroblasts in each group were analyzed by RT‐qPCR. **(D‐E)** Western blot analysis of TGFβ, Smad2, p‐Smad2, Col1a1, and Col3a1 in each group fibroblasts. **(F)** Immunofluorescent staining for Col1a1 in each group fibroblasts. Results represent mean ± SEM, ***p < 0.001, **p < 0.01, *p < 0.05, n = 3.

CellChat analysis showed that fibroblasts had the highest number and intensity of differential cellular interactions (Figure S5B–D). The communication strength between macrophages and fibroblasts was significantly enhanced after testicular Cd exposure (Figure 7A). Thus, 3T3‐L1 mice embryonic fibroblasts were treated with the culture supernatant from the aforementioned macrophage to explore cellular interactions (Figure 7B). The cell culture supernatant of macrophages treated with Cd‐EV or Cd‐CS upregulated the protein and partial mRNA expression of fibrosis‐related markers TGFβ, p‐Smad2, Col1a1, and Col3a1 in fibroblasts (Figure 7C–E). Similar result was obtained when Col1a1 was analyzed by immunofluorescence staining (Figure 7F). These findings suggest that activated macrophages lead to the activation of the TGFβ pathway in fibroblasts, resulting in fibrosis.

### 2.8 Cd‐Exposed Sertoli Cell‐Derived EVs Induced an Increase in Inflammatory Factors and Collagen in the Testis

**FIGURE 8:**
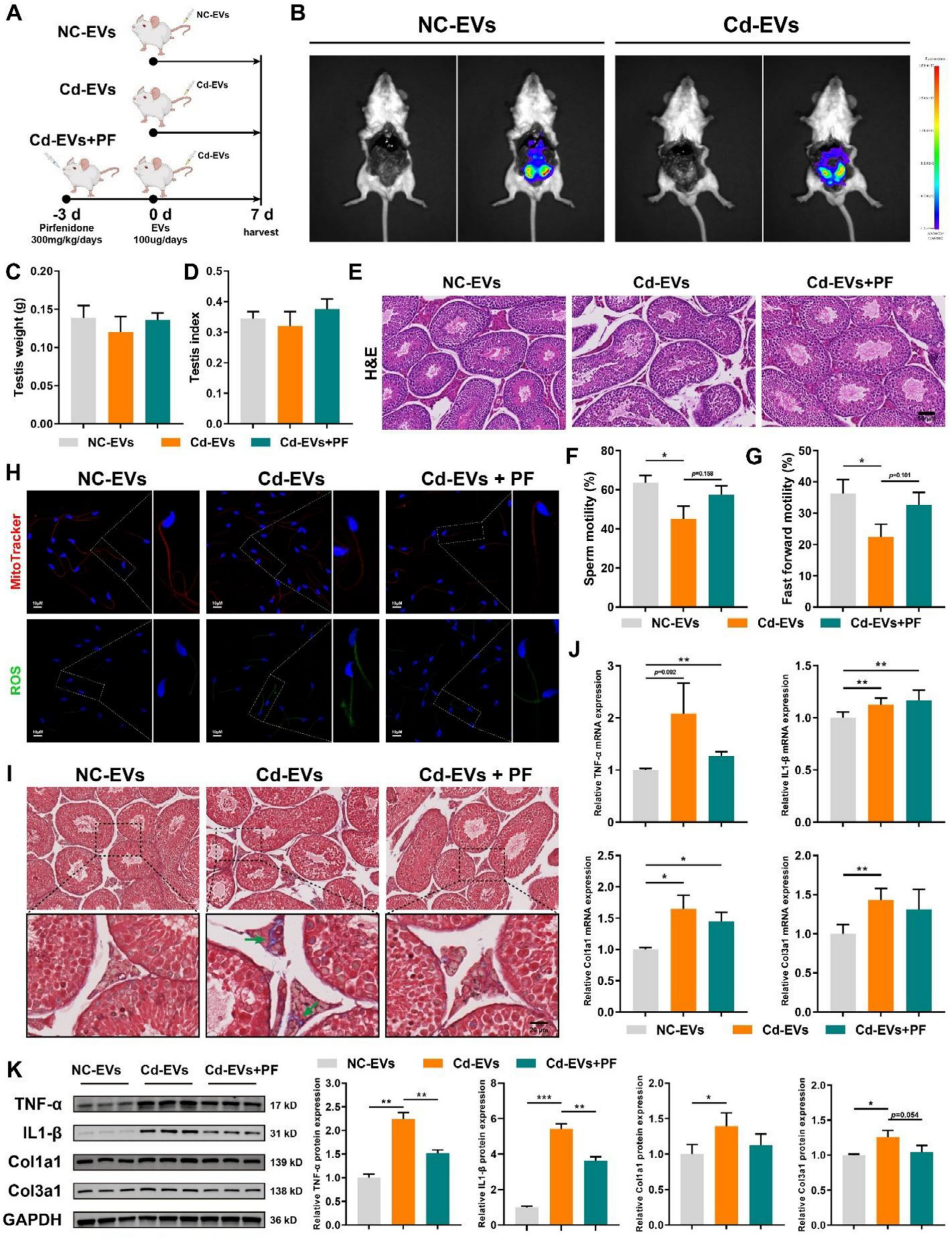
Cd‐exposed Sertoli cell‐derived EVs trigger inflammation and collagen increase in the testis. **(A)** A schematic diagram of the mice treatment administration process. Negative control extracellular vesicles, NC‐EVs. Cd‐exposed Sertoli cell‐derived extracellular vesicles, Cd‐EVs. Cd‐exposed Sertoli cell‐derived extracellular vesicles and pirfenidone treatment, Cd‐EVs + PF. **(B)** The distribution of EVs were detected with In‐Vivo Imaging System. **(C)** Comparison of testis weights. **(D)** Testis index in each group. **(E)** Testicular sections stained with H&E. **(F)** The sperm motility in mice epididymal. **(G)** The percentage of the fast forward sperm. Results represent mean ± SEM, *p < 0.05, n = 6. **(H)** Staining of sperm mitochondria using MitoTrack probe. Reactive oxygen species (ROS) detected by DCFH‐DA. **(I)** Masson staining of testis sections. **(J)** TNF‐α, IL‐1β, Col1a1, and Col3a1 mRNA detection in the testis by RT‐qPCR. **(K)** Western blot analysis and quantification for TNF‐α, IL‐1β, Col1a1, and Col3a1 in the testis. Results represent mean ± SEM, ***p < 0.001, **p < 0.01, *p < 0.05, n = 3.

Based on the above results, we investigated the role of Sertoli cell‐derived EVs in testicular fibrosis induced by Cd exposure. Mice were injected with Cd‐EVs and NC‐EVs, respectively, via the tail vein. As displayed in experimental design diagram, a group of mice received oral administration of the anti‐inflammatory and anti‐fibrotic molecule pirfenidone before being injected with Cd‐EVs (Figure 8A). Fluorescence images were taken with the animal imaging system to evaluate the specificity and body distribution of the DiR‐labeled EVs. The prepared Sertoli cell‐derived EVs were detected in internal organs and were enriched in the epididymal fat and testis (Figure 8B). After EVs injection, the testis weight and testis index did not differ significantly among the groups (Figure 8C,D). H&E staining revealed that compared to the NC‐EVs group, the seminiferous tubules of Cd‐EVs‐treated mice exhibited sparser cellular architecture and a decreased number of spermatozoa (Figure 8E). A reduction in epididymal sperm motility and movement capacity was detected in the Cd‐EVs group using CASA system (Figure 8F,G). Cd‐EVs injection led to decreased mitochondrial membrane potential and increased ROS levels in sperm (Figure 8H). Pirfenidone partially relieved these negative effects of Cd‐EVs on mice sperm (Figure 8F‐H). Sirius Red staining revealed increased collagen fibers in the testicular interstitium of Cd EVs group mice (Figure 8I). Western blot and RT‐qPCR analysis showed increased expression of TNF‐α, IL‐1β, Col1a1, and Col3a1 in the testis of Cd‐EV‐injected mice (Figure 8J,K). To further investigate whether Cd‐EVs activate macrophages and fibroblasts in vivo, we performed immunofluorescence staining on testis sections from mice injected with NC‐EVs or Cd‐EVs. Immunofluorescence analysis revealed that NF‐κB‐p65 nuclear translocation was predominantly localized to F4/80‐positive macrophages following Cd‐EVs treatment (Figure S7AB), confirming macrophage‐specific activation of the NF‐κB pathway. Additionally, enhanced TGF‐β signaling was observed in Col3a1‐positive fibroblasts (Figure S7C,D), indicating fibroblast‐specific activation of the TGF‐β pathway. These results indicated that Cd‐EVs caused testicular inflammation and fibrosis, and pirfenidone alleviated these effects.

As shown in Figure S8A, mice were pretreated with TAK‐242 (3 mg/kg/day, i.p.) for three consecutive days prior to Cd exposure. The results demonstrated that TAK‐242 administration significantly restored Cd‐induced testicular weight loss (Figure S8BC). Western blot analysis confirmed that TAK‐242 effectively inhibited TLR4 protein expression in the testis (Figure 8D). Furthermore, TAK‐242 treatment markedly suppressed the Cd‐induced upregulation of inflammatory cytokines TNF‐α and IL‐1β, as well as the fibrotic markers Col1a1 and Col3a1 (Figure S8D,E).

## 3 Discussion

Cadmium exposure is a well‐documented risk factor for male reproductive toxicity, associated with direct oxidative stress and apoptosis in testicular cells [8, 17]. However, the intricate cell–cell communication mechanisms orchestrating the resultant inflammation and tissue fibrosis remain poorly understood. Here, we unveil a novel pathway wherein cadmium induces various forms of programmed cell death (PCD) in Sertoli cells, prompting them to release extracellular vesicles (EVs) loaded with damage‐associated molecular patterns (DAMPs) and mitochondrial fragments. These Cd‐EVs are subsequently internalized by testicular macrophages, triggering their pro‐inflammatory activation via the TLR4/NF‐κB signaling pathway. This inflammatory cascade activates fibroblasts and promotes collagen deposition, ultimately leading to testicular fibrosis. Our study thus identifies Sertoli cell‐derived EVs as critical mediators in propagating cadmium‐induced injury signals, establishing a crucial link between initial cellular damage and the resultant inflammatory and fibrotic microenvironment in the testis.

Our in vivo findings unequivocally demonstrate that acute cadmium exposure leads to severe testicular injury and impaired spermatogenesis, corroborating the well‐established reproductive toxicity of this heavy metal. Zhou et al. reported that consecutive 7‐day intraperitoneal injections of 3.0 mg/kg CdCl_2_ induced significant testicular injury in mice, characterized by pyroptosis, oxidative stress, and impaired sperm quality [9]. Similarly, single intraperitoneal injection of 1.2 or 2 mg/kg CdCl_2_ causes testicular injury, including germ cell apoptosis and seminiferous epithelial degeneration [18, 19]. Based on this evidence, we selected a single intraperitoneal dose of 1.5 mg/kg CdCl_2_ to construct our acute testicular injury model. We observed a progressive accumulation of Cd in the testis, a significant decrease in testis weight, and severe macroscopic alterations including tissue hardening and adhesion, consistent with previous reports [9, 20]. Histologically, the model was validated by pronounced seminiferous tubule atrophy, a widened interstitial space filled with a collagen matrix, a drastic reduction in sperm count with abnormal morphology, and a marked decrease in the germ cell marker DDX4. At the ultrastructural level, swollen mitochondria with disrupted membranes provided further evidence of Cd‐induced cellular injury. Collectively, these pathological outcomes confirm the successful establishment of a robust and reproducible model of acute CdCl_2_‐induced testicular injury in mice using a single 1.5 mg/kg dose.

Indeed, it has already been reported that Cd exposure induces testicular injury through the activation of multiple forms of programmed cell death, including apoptosis, autophagy, necrosis, pyroptosis, ferroptosis. Our multi‐omics data provide a comprehensive map of the molecular disruptions in Cd‐induced testicular injury. Bulk transcriptomic analysis not only confirmed the involvement of classical apoptosis but also revealed a significant upregulation of genes associated with autophagy, necroptosis, pyroptosis, and ferroptosis, with cuproptosis being a notable exception that was not significantly enriched. Apoptosis is a central mechanism, as Cd exposure increases testicular germ cell and Leydig cell apoptosis via mitochondrial dysfunction, oxidative stress, and activation of caspase pathways, leading to impaired spermatogenesis and reduced fertility [17, 21]. Autophagy, while initially protective by clearing damaged organelles, becomes dysregulated under Cd stress [22]. Excessive or defective autophagy amplifies cell death and testicular damage, and its inhibition exacerbates Cd‐induced apoptosis [23]. Pyroptosis, an inflammatory form of programmed cell death, is also triggered by Cd in testicular tissue through the activation of the AIM2 inflammasome and increased oxidative stress, resulting in elevated expression of pyroptosis‐related proteins such as GSDMD, Caspase‐1, and IL‐1β, and contributing to germ cell loss and inflammation [9, 24]. Ferroptosis, characterized by iron‐dependent lipid peroxidation, is promoted by Cd through disruption of iron homeostasis, increased lipid peroxides, and inhibition of antioxidant defenses, further impairing testosterone synthesis and testicular function [25]. The convergence of transcriptomic and proteomic data strongly argues that the simultaneous induction of multiple PCD forms is a fundamental characteristic of Cd toxicity, leading to the subsequent disruption of the spermatogenic niche.

Single‐cell RNA sequencing (scRNA‐seq) provides a powerful tool for investigating testicular development and reproductive disorders [26, 27]. Mammalian testis function depends on the presence of characteristic cell types, including germ cells and somatic cells. Testicular somatic cells, including Sertoli cells and Leydig cells, provide a stem cell niche and microenvironment for spermatogenesis [28]. Leydig cells, responsible for testosterone production, show reduced activity of steroidogenic enzymes after Cd exposure. Cd causes mitochondrial disorganization, increased lipid peroxidation, and apoptosis in Leydig cells [21]. For Sertoli cells, Cd exposure leads to mitochondrial alterations, disruption of the blood‐testis barrier, and downregulation of key gene involved in Sertoli cell function [29]. It is reported that Cd exposure may stimulate the proliferation and dysfunction of the mural cells and endothelial cells of blood vessels, which may lead to abnormal function of the testis [30]. However, previous studies have generally focused only on the effects of Cd on a single cell type and lack the context of the complex tissue microenvironment. This study provides comprehensive landscapes of the transcriptional network of cells in Cd‐exposed testis using scRNA‐seq method. Our results of single‐cell analysis reveal the number of Sertoli cells decreased sharply, while a dramatic increase in the fibroblast population. The results were further confirmed by immunofluorescence analysis. Fibroblasts are a cell type that was previously underestimated in Cd induced testicular injury. Based on the macroscopic phenotype of Cd‐exposed testis, we speculate that excessive proliferation of fibroblasts leads to testicular fibrosis. Subsequently, augmented collagen deposition was indicated by Masson and Sirius staining in Cd‐exposed testis. Western blot analysis further confirmed the fibrotic development in Cd‐exposed testis. Our analysis not only confirmed a massive depletion of germ cells but also revealed a profound reconfiguration of the testicular somatic cell landscape following Cd exposure.

scRNA‐seq data reveal that Sertoli cells are the most susceptible somatic cell type in cadmium‐induced testicular damage. The in vitro model definitively demonstrated that Cd exposure directly triggers a multi‐modal PCD response in Sertoli cells, including apoptosis, necroptosis, pyroptosis, and ferroptosis, which was associated with mitochondrial dysfunction and ROS overproduction. This has been demonstrated previously [22, 31]. Sertoli cells provide structural and nutritional support for developing germ cells, regulate the testicular microenvironment, and promote spermatogenesis [32]. Importantly, our scRNA‐seq analysis revealed that the differentially expressed genes (DEGs) in Sertoli cells after Cd exposure were significantly enriched in the regulation of exosomal secretion. This seems contradictory to previous studies, which reported a Cd‐induced reduction in exosomal secretion linked to sperm maturation [33]. However, this previous study primarily focused on the multivesicular body pathway and small, nano‐scale CD63‐positive exosomes that are crucial for normal intercellular communication. Extracellular vesicles (EVs) are a highly heterogeneous group of membrane‐bound particles. It is reported that PCD affects the production of extracellular vesicles. During apoptosis, cells undergo membrane blebbing and fragmentation, producing large apoptotic bodies and smaller apoptotic microvesicles [34]. In necroptosis, MLKL interacts with endosomal sorting complexes (ESCRT) to facilitate EV formation [35]. Therefore, our data indicate that Cd induces PCD in Sertoli cells and exacerbates the release of EVs.

Sertoli cells‐derived EVs in response to Cd exposure were further investigated. Proteomic profiling of these EVs revealed a specific enrichment of proteins linked to the PCD pathways activated in the cells, including necroptosis and ferroptosis, which is consistent with our scRNA‐seq data. DAMPs, which are endogenous danger molecules, are released from damaged or dying cells during cellular stress [36]. We identified a significant load of DAMPs within these EVs, most notably heat shock protein. Heat shock proteins (HSPs) maintain protein homeostasis and protect cells under stress [37]. When released extracellularly due to cell damage or stress, HSPs have been proposed to act as DAMPs [38]. In addition, the expression of DAMPs was also significantly increased both in cells and in the cell culture supernatant after Cd exposure, which supported the results found in the EVs. Interestingly, the proteomics data of EVs suggest that mitochondrial components are particularly prominent. The enhanced colocalization between the mitochondrial protein HSP60 and the late endosome marker Rab7 in Cd‐exposed Sertoli cells offers a mechanistic insight into the biogenesis of DAMPs and mitochondria‐containing EVs. This observation suggests that damaged mitochondria may be targeted for degradation through mitophagy, but instead of complete lysosomal clearance, mitochondrial contents intersect with the endosomal pathway [39, 40]. Specifically, mitochondria‐derived vesicles or mitophagosomes may fuse with multivesicular bodies, leading to the packaging of mitochondrial components and associated DAMPs into intraluminal vesicles for subsequent release as exosomes or small EVs [41]. This process, sometimes referred to as ‘mitochondrial EV’ biogenesis, has been increasingly recognized as a mechanism for cells to eliminate damaged mitochondrial cargo while simultaneously generating signaling vesicles that can influence recipient cell behavior. Mitochondrial proteins, as important components of EVs, have been reported to play a role in cellular communication [42]. Future studies investigating the molecular machinery governing this endosome‐mitochondria crosstalk, such as the role of specific ESCRT components or Rab GTPases, would further elucidate how stressed Sertoli cells package and release these bioactive EVs.

CellChat analysis revealed that intercellular communication in the Cd‐exposed testis was enhanced in both number and intensity. Especially, the intercellular communication between Sertoli cells and macrophages, as well as between macrophages and fibroblasts, was enhanced. We speculate that Sertoli cell‐derived EVs activate macrophages and lead to aberrant proliferation of fibroblasts. Both the conditioned medium and the isolated EVs from these Cd‐exposed Sertoli cells were sufficient to polarize macrophages toward a pro‐inflammatory M1 phenotype, as evidenced by the significant upregulation of TNF‐α and IL‐1β mRNA and the increased surface expression of the activation marker CD86. Mechanistically, we identified the TLR4/NF‐κB pathway as the central signaling axis mediating this response. Multiple studies have demonstrated that EVs from various cell types can induce macrophage polarization via this signaling axis [43]. Studies have shown that DAMPs, particularly HSPs and mitochondrial proteins, are key EV cargoes that activate macrophages via TLR4/NF‐κB [44, 45]. In a mouse model of alcoholic liver disease, circulating EVs induce macrophage activation, and HSP90 within these EVs is identified as the key mediator [44]. These results supported our findings. Beyond the identified DAMPs and mitochondrial components, the enrichment of necroptosis and ferroptosis‐related proteins in Cd‐EVs raises intriguing possibilities. These EV‐associated proteins could potentially modulate macrophage function through mechanisms distinct from TLR4 activation. Future studies exploring the functional contribution of these specific protein cargoes to macrophage activation would further illuminate the complexity of EV‐mediated intercellular communication in cadmium‐induced testicular injury. Activated macrophages play a central and dynamic role in the development and progression of fibrosis across multiple organs [46, 47]. Upon tissue injury, macrophages undergo phenotypic changes and secrete a variety of mediators that directly and indirectly influence fibroblast activation and extracellular matrix (ECM) remodeling [48]. The conditioned medium from these activated macrophages significantly upregulates fibrosis markers in fibroblasts. In myocardial fibrosis, M1 macrophages show high expression of cytokine IL‐1β, which can activate fibroblasts [49]. In vitro inhibition of IL‐1β suppressed fibroblast activation, further confirming the pivotal contribution of M1 macrophages and their release of IL‐1β to the pathogenesis of diabetic cardiomyopathy [49]. This is similar to our experimental results. To further verify if these Sertoli cell‐derived EVs are associated with testicular fibrosis, we conducted in vivo studies using EVs. The results showed that Cd exposure induced EVs led to an increase in collagen levels in the testis and impaired sperm quality. It is important to acknowledge the inherent limitations regarding EV dose selection in this study. Accurately mimicking the physiological concentrations of Sertoli cell‐derived EVs in the testicular interstitial space following Cd exposure remains technically challenging. While our dose‐ranging pilot experiments established 100 µg/day as the minimal dose required for consistent testicular detection, and the inclusion of NC‐EVs as a control group supports the specificity of Cd‐EV effects, we cannot exclude the possibility that the EV concentrations used may exceed physiological levels. Future advances in EV quantification technologies and more sophisticated delivery approaches may enable more precise recapitulation of endogenous EV signaling dynamics. Despite this limitation, the concordance between our in vitro dose‐response data and the in vivo observations, together with the cell type‐specific effects confirmed by immunofluorescence, supports the biological relevance of our findings.

In conclusion, our study utilized multiple omics to elucidate a novel pathogenic axis in cadmium‐induced testicular injury, revealing a complex intercellular communication network. We demonstrate that cadmium triggers a multifaceted programmed cell death in Sertoli cells, which in turn release a distinct population of EVs loaded with DAMPs and mitochondrial components. These EVs are internalized by macrophages, activating them via the TLR4/NF‐κB pathway to secrete pro‐inflammatory cytokines. This inflammatory milieu subsequently activates fibroblasts, promoting a pro‐fibrotic response through the TGF‐β/Smad pathway and leading to testicular fibrosis. While direct cytotoxicity of Cd on germ cells and BTB integrity represents the primary insult, our findings unveil an amplification mechanism wherein stressed Sertoli cells release EVs that activate macrophages and fibroblasts, propagating inflammation and fibrosis. These two pathways are likely interconnected: direct cellular damage triggers EV release, and the ensuing fibrotic microenvironment further impairs germ cell survival and function, creating a vicious cycle that culminates in severe testicular injury. Our findings not only deepen the understanding of environmental toxicant‐induced male infertility but also highlight potential therapeutic targets for intervening in the progression of testicular fibrotic disease.

## 4 Materials and Methods

### 4.1 Ethical Statement

The experimental procedures performed in mice followed the guidelines of the Ethics Committee of Sichuan Agricultural University (SICAU) and were approved by the Ethics Committee of SICAU (2023102011).

### 4.2 Animal Models and Experiments

All procedures involving animals were reviewed and approved by the Sichuan Agriculture University ethics committee. Adult male ICR mice (10 week‐old) were supplied by the CHENGDU DOSSY EXPERIMENTAL ANIMALS CO., LTD. (Chengdu, China). All mice were allowed to feed and drink freely, and were maintained within a temperature and humidity range of 20°C–25°C and 50%–60%. CdCl_2_ was dissolved in saline to prepare a working solution at a concentration of 0.3 mg/mL. The mice received a single intraperitoneal injection of 1.5 mg/kg CdCl_2_ to construct the testicular injury model (Cd, n = 10). Mice in the control (Con, n = 10) group received normal saline injection. After 7 days of injection, the mice were sacrificed, and testis and epididymis tissues were collected for analysis. In this study, the Cd dosage was chosen based on the results of multiple previous studies [9, 20, 50]. One side of the collected epididymis was placed in PBS at 37°C and incubated for 10 min. For morphological examination of sperm, 10 µL of the sperm suspension was taken to prepare a sperm smear for observation under a microscope. Sperm motility parameters were measured using a computer‐assisted sperm analysis (CASA) system (Minitube, Germany).

For EV treatment experiments, mice were injected via the tail vein with 100 µg of Sertoli cell‐derived EVs daily for 7 consecutive days. The male ICR mice were divided into three groups (n = 6 per group): (1) NC‐EVs group: mice received EVs isolated from control Sertoli cells; (2) Cd‐EVs group: mice received EVs isolated from Cd‐exposed Sertoli cells; (3) Cd‐EVs + PF group: mice were pretreated with pirfenidone (300 mg/kg/day) by intraperitoneal injection for 3 consecutive days prior to Cd‐EV injection, followed by 7 days of Cd‐EV administration.

For TLR4 inhibition experiments, mice were randomly assigned to three groups (n = 6 per group): (1) DMSO control group: mice received vehicle (DMSO) intraperitoneally followed by saline injection; (2) Cd + DMSO group: mice received DMSO pretreatment followed by a single intraperitoneal injection of CdCl_2_ (1.5 mg/kg); (3) Cd + TAK‐242 group: mice were pretreated with TAK‐242 (3 mg/kg/day, i.p.) for 3 consecutive days prior to CdCl_2_ injection (1.5 mg/kg). All mice were sacrificed 7 days after Cd exposure for sample collection.

### 4.3 Histological Analysis

Testis was fixed overnight in 4% (w/v) PFA, dehydrated, embedded in paraffin, and cut into 5 µm sections. Testis sections were treated with hematoxylin and eosin (H&E) staining for the observation of morphology. For Masson staining, interstitial collagen deposition in the testis tissues was stained using a Masson staining kit (G1346, Solarbio, China). Sirius red staining was performed using Modified Sirius Red Stain Kit (G1472, Solarbio, China)

### 4.4 Plasma Hormones Test

Blood samples were collected into anticoagulant tubes and centrifuged at 3000 × g for 15 min at 4°C to obtain plasma. The levels of follicle‐stimulating hormone (FSH), luteinizing hormone (LH), and testosterone in the plasma were measured using ELISA kits (BY‐WJZF1596, BY‐WJZF1658, BY‐WJZF0053, Byabscience Biotechnology Co., Ltd) according to the manufacturer's instructions. The absorbance was measured at 450 nm using a microplate reader.

### 4.5 Cell Culture and Treatment

The mouse Sertoli cells (TM4, CL‐0456), macrophage cell line (RAW 264.7, CL‐0190), and fibroblast cell line (3T3‐L1, CL‐0006) were purchased from Procell Life Science and Technology Co., Ltd. (Procell, China). Cells were verified to be free of mycoplasma contamination using the GMyc‐PCR Mycoplasma Test Kit (40601ES20, Yeasen, China). Sertoli cells were cultured in DMEM (Gibico, Shanghai, China) supplemented with 5% Horse Serum (F830‐100, BaiDi Biotechnology Co., Ltd.(BDBIO)) and 2.5% FBS (ExCell Bio, China). Other cells were cultured in DMEM supplemented with 10% FBS and 1% penicillin and streptomycin at 37°C with 5% CO2. All cell lines were confirmed to be free of mycoplasma contamination before the study.

For Sertoli cells cytotoxicity assay, cells were seeded in 96‐well plates at 2.0 × 10^3^ cells/well. Cells were treated with CdCl_2_ at different concentrations when reaching 90% confluency. After 24 h of treatment, the CCK‐8 assay (C0005, TargetMol, USA) was used to evaluate the cell viability. Sertoli cells were seeded in 12‐well plates and, once reaching 90% confluency, were treated with CdCl_2_ for 24 h before being harvested for RNA or protein analysis.

After being treated with Cd for 24 h, the Sertoli cells were washed with PBS, and the medium was replaced. After 24 h, the supernatant was collected and centrifuged to remove cellular debris. RAW264.7 macrophages were treated by replacing half of the culture medium with the collected Sertoli cell‐conditioned supernatant or adding 50 ug/mL Sertoli cell‐derived extracellular vesicles (described later). After 24 h of treatment, the macrophages were harvested for subsequent experiments. The aforementioned macrophages were re‐cultured in fresh medium for another 24 h. The macrophage culture supernatant was collected and centrifuged to remove cellular debris. 3T3‐L1 fibroblasts were treated by replacing half of the culture medium with the collected macrophage‐conditioned supernatant. After 24 h culture, 3T3‐L1 cells were used in the subsequent experiments.

### 4.6 RT‐qPCR

Total RNA was extracted with the Trizol reagent from NCTC1469 cells. Then, the total RNA was reverse transcribed into cDNA using a Takara PrimeScript II 1st strand cDNA Synthesis Kit (Takara). qPCR was performed using Universal SYBR qPCR Master Mix (Vazyme). The relative gene expressions were calculated using the 2−ΔΔCt method.

### 4.7 Mitotracker and ROS Staining

MitoTracker Deep Red FM (S29947, Yuanye, Shanghai, China) were resuspended in DMSO to make a 1 mm stock. The stain was further diluted in DMEM to a working concentration of 100 nm. Sertoli cells were treated with CdCl_2_ for 24 h and then incubated with MitoTracker working solution for 20 min. Cells were observed by fluorescence microscopy. For MitoTracker staining of sperm, MitoTracker was added to the sperm suspension at a final concentration of 100 nm and incubated in the dark for 10 min. After staining, sperm smears were prepared immediately and observed under a fluorescence microscope. DCFH‐DA (ID3130, Beijing Solarbio Science & Technology Co., Ltd.) was performed to measure ROS levels. Similar to MitoTracker staining, Sertoli cells and sperm were incubated with a 10 µm ROS probe for 30 min. Nuclei staining was done with Hoechst 33258 (CB2761, Coolaber, China)

### 4.8 Extracellular Vesicles Isolation and Characterization

To isolate extracellular vesicles derived from Sertoli cells, the cells were seeded in 10‐cm culture dishes (803100B, CellPro Biotechnology). When the cells reached 90% confluency, the culture medium was removed and replaced with DMEM supplemented with 10% exosome‐depleted fetal bovine serum (NZF‐ED‐500, UPSILON, USA). After 24 h of culture, EVs from culture supernatant were extracted by a standard differential centrifugation protocol. In brief, culture supernatant was serially centrifuged at 300 × g for 10 min, 3000 × g for 10 min, and 10 000 × g for 20 min at 4°C to remove cells, debris, and large vesicles. The supernatant was passed through 0.22 µm pore PES filters (Millipore) and then centrifuged at 120 000 × g for 90 min at 4°C to pellet EVs. Finally, EVs were washed once and resuspended with PBS. The protein concentration in the EV was measured by BCA assay kit (AP12L025, Life‐iLab, China). The sizes of EVs were measured using the NanoSight NS300 system (NanoSight, UK). The purified EVs were diluted 1:100–1:500 in PBS and subjected to nanoparticle tracking analysis. Particle numbers were analyzed with the Nanoparticle Tracking Analysis (NTA) 3.0 software.

### 4.9 Extracellular Vesicles Uptake

EVs were labelled using the PKH67 green fluorescent dye (UR52303, Umibio, China) following the manufacture's protocol. In brief, EVs were stained with PKH67 using a working solution prepared by mixing the PKH67 linker with Diluent C at a 1:9 ratio in the dark at room temperature. Unbound dye was removed by ultracentrifugation (100 000×g, 30 min, 4°C). The labeled EVs were incubated on RAW 264.7 cells for 24 h at 37°C, and cells were washed with 1 × PBS. Cells were then fixed, permeabilized, and followed by staining with Aluor594‐conjugated phalloidin (KGE2413, KeyGene BioTECH, China) and DAPI (AS21L123, Life‐iLab, China).

### 4.10 Flow Cytometry

Macrophages were treated with Sertoli cell‐derived EVs or culture supernatant for 24 h and subsequently harvested for flow cytometry analysis. Cells were collected and resuspended in 100 µL of PBS. The samples were incubated with APC‐conjugated anti‐CD86 (130‐122‐130, Miltenyi Biotec) and PE‐conjugated anti‐CD206 (130‐123‐168, Miltenyi Biotec) antibody at room temperature for 30 min. After washing with 1× PBS, the cells were analyzed by flow cytometry to examine the expression levels of CD86 and CD206.

### 4.11 Transmission Electron Microscopy

Testis tissues of mice and Sertoli cells were harvested and fixed in 2.5% glutaraldehyde overnight at 4°C. After postfixation in 1% OsO4 followed by uranyl acetate, the samples were gradiently dehydrated in acetone (50%, 70%, 90%, and 100% separately) and embedded in epoxy resins. Ultrathin sections were collected on formvar‐coated grids and stained with uranyl acetate and lead citrate, and then, the samples were examined with a Hitachi HT7800 transmission electron microscope. EVs were fixed with 2.5% glutaraldehyde for 2 h at 4°C. A volume of 10 µL of an EV sample was applied to a copper mesh and allowed to stand for 5 min. Excess liquid was absorbed using filter paper. After being blotted and air‐dried, the samples were stained with 10 µL 2% uranyl acetate for 1 min. EVs were visualized using an HT7800 transmission electron microscope.

### 4.12 In Vivo Fluorescence Imaging

Imaging system AniView 100 (Biolight, China) was used to observe mice at 3 different time points (24 h, 3, 7 day) after the CdCl2 treatment. According to the manufacturer's instructions, Leadmium Green AM dye was dissolved in DMSO and then diluted with saline to prepare the working solution. Mice were intravenously injected via the tail vein with Leadmium Green AM dye and imaged two hours later to observe the distribution of cadmium in the testes.

To track Sertoli cell‐derived EVs in vivo, EVs were labelled with 2 µm DiR (FD‐CDR003, Biolight, China) for 1 h at 37°C and then purified by ultracentrifugation (100 000 × g, 30 min, 4°C). The mice were injected with DiR‐labeled EVs via tail vein. At 24 h after the injection, the mice were imaged using AniView 100 Imaging System (BLT, China).

### 4.13 Immunohistochemistry and Immunofluorescence Staining

For immunohistochemistry, mouse testis tissues were fixed in 4% paraformaldehyde, embedded in paraffin, and sectioned. Following deparaffinization, antigen retrieval was performed with sodium citrate buffer. Sections were incubated with anti‐DDX4 antibody (WL03040, Wanleibio, China) overnight at 4°C, followed by horseradish peroxidase‐coupled secondary antibody for 30 min at room temperature. The bound antibody was visualized using a DAB kit. For immunofluorescence staining of testis tissues, Sections were incubated with anti‐SOX9 antibody (R380995, Zenbio, China), anti‐CYP17A1 antibody (R24045, Zenbio, China), anti‐ FN1 antibody (250073, Zenbio, China), anti‐F4/80 antibody (BYab‐17721, BYabscience, China), anti‐CD86 (BYab‐14107, BYabscience, China) using TSA Plus Fluorescence Kit (RC0086plus, Huilanbio, China) according to manufacturer's protocol.

For immunofluorescence staining of cells, cells were washed with PBS and fixed with 4% paraformaldehyde for 10 min at room temperature. Then, cells were permeabilized with 0.1% Triton X‐100 for 5 min at room temperature, blocked with BSA 5%, and incubated with primary antibodies overnight at 4°C. The cells were stained with fluorescent‐conjugated secondary antibodies, and the nuclei were stained with DAPI for 5 min. The primary and secondary antibodies include: anti‐Rab7 (R25524, Zenbio, China), anti‐HSP60 (201060, Zenbio, China), anti‐TLR4 (BYmab‐17807, BYabscience, China), anti‐NF‐κB p65 (WL01980, Wanleibio, China), anti‐Col1a1 (HA722517, HUABIO, China), FITC‐Goat Anti‐Rabbit IgG H&L (WLA032, Wanleibio, China), TRITC‐Goat Anti‐Mouse IgG H&L (511102, Zenbio, China), TRITC‐Goat Anti‐Rabbit IgG H&L (511202, Zenbio, China). The colocalization between the channels of interest was then measured using the Colocalization Finder plugin in Fiji, and Pearson's correlation coefficient was calculated.

### 4.14 RNA Sequencing and Transcriptome Analysis

RNA sequencing (RNA‐seq) and transcriptomic analysis were performed as described previously [51]. Testis tissues were harvested into Trizol to isolate RNA. Library construction and sequencing were completed by Novogene (Beijing, China). The libraries were sequenced on the DNBSEQ‐T7 platform. Data quality was assessed by the FastQC tool after removal of adaptor sequences, ambiguous “N” nucleotides (proportion of “N” > 5%), and low‐quality sequences (quality score < 10). Reads were mapped to the Mus musculus GRCm39 reference genome using HISAT2, SAMtools, and StringTie. Differentially expressed genes were identified using DESeq2. Differentially expressed genes were identified using DESeq2 v1.32.0 with the cutoff: FDR < 0.05, |log2 fold change| > 1.

### 4.15 Functional Enrichment Analysis

GO enrichment, KEGG pathway analysis of differentially expressed genes or proteins were carried out DAVID (https://david.ncifcrf.gov/). Gene set enrichment analysis (GSEA) was performed using GSEA v3.0 (http://www.broadinstitute.org/gsea/). Data visualization was carried out using OmicStudio (https://www.omicstudio.cn/tool).

### 4.16 Proteomic Analysis

The quantitative proteomics of testis tissue was performed by Novogene Co., Ltd (Beijing, China) to identify differential protein expression profiling. Briefly, testis samples (n = 3 per group) were homogenized in SDS lysis buffer and quantified using the BCA assay kit. Each protein sample was taken and digested at 37°C for 4 h. Formic acid was mixed with digested sample, adjusted pH under 3, and centrifuged at 12 000 g for 5 min at room temperature. The supernatant was slowly loaded to the C18 desalting column, washed with washing buffer. The eluents of each sample were collected and lyophilized. LC‐MS/MS analysis was performed on a Vanquish Neo UHPLC system equipped with a C18 pre‐column (5 mm × 300 µm, 5 µm) and a C18 analytical column (150 µm × 15 cm, 2 µm) heated at 50°C, coupled to a Thermo Orbitrap Astral mass spectrometer with an Easy‐spray ESI source (2.0 kV, 290°C). The raw files were searched and analyzed using the DIA‐NN library search software, according to the protein database.

Proteomic analysis of Sertoli cell‐derived EVs was completed by Umibio Biotechnology Co., Ltd (Shanghai, China). 10 µg of extracted proteins from each sample was then subjected to SISPROT. Peptides were separated on a Vanquish Neo upgraded UHPLC with a C18 pre‐column of 174500 (5 mm × 300 µm,5 µm, thermo). All RAW files were analyzed using the DIA‐NN (v1.8.1, https://github.com/vdemichev/DiaNN). MS2 spectra were searched against the UniProtKB proteome database containing both Swiss‐Prot reference protein sequences. The original search result data was median normalized.

### 4.17 Single‐Cell RNA Sequencing Analysis

Testes from three control and three cadmium‐exposed mice were respectively pooled to create two composite samples for single‐cell suspension preparation. Single cell suspension preparation and sequencing were performed by Novogene (Beijing, China). The cells of each sample were then processed with the MobiCube High Throughput Single Cell 3' RNA‐Seq Kit (v2.1) as per manufacturer's instruction in Novogene Bioinformatics Technology Co., Ltd (Tianjing, China). Briefly, the cells were loaded into microfluidic chip of Chip A Single Cell Kit (S050100201, MobiDrop) to generate droplets with MobiNova‐100 (A1A40001, MobiDrop), encapsulating individual cells with barcoded gel beads for sequencing. Single‐cell RNA‐seq library preparation was performed using the MobiDrop platform, with droplets generated and light‐cleaved by the MobiNovaSP‐100 (A2A40001, MobiDrop). After mRNA capture, barcoding, and reverse transcription, libraries were constructed with the High Throughput Single Cell 3'RNA‐Seq Kit v2.0 (S050200201, MobiDrop) and 3' Single Index Kit (S050300201, MobiDrop), and sequenced on an Illumina NovaSeq system with 150‐bp paired‐end reads in Novogene.

Single‐nucleus RNA‐seq data were processed as follows: Raw reads (Read1: barcode/UMI; Read2: cDNA fragment) were quality‐controlled using Fastp [52]. Subsequently, reads were aligned to the mouse reference genome (GRCm39) and a gene‐cell matrix was generated using MobiVision v2.0 with default parameters (https://www.mobidrop.com/bioinformatics‐analysis‐software/mobivision‐news/software‐download). The gene‐cell matrix from MobiVision was loaded into the Seurat package(v4.4.0) [53]. Cells were filtered based on the following thresholds: 200–6000 genes, 500–15 000 UMIs, and mitochondrial/hemoglobin gene ratios below 20% and 5%, used scDblFinder [54] to remove doublets, respectively, yielding 48,881 high‐quality cells. Data were normalized, and 2000 highly variable genes were selected for PCA (30 PCs). Harmony was applied for batch correction [55]. Then clusters were identified using the Seurat function ‘FindClusters’ with appropriately selected PCs and ‘resolution = 0.3 and 0.5’. The single‐cell data structures and trajectories were separately visualized and explored by UMAP. Based on the marker genes of each cell type and cluster‐enriched genes [56, 57, 58]. We identified the cell type of each cluster. Seurat function FindMarkers was used to identify differentially expressed genes in the clusters, and the Wilcox test was used. The detection parameters for differentially expressed genes in the clusters were pct1 > 0.25, pct2 > 0.25, and |avg_log2FC|>1, and p value<0.05. The CellChat R package (v1.6.1) was used to infer the cell‐cell interactions [59]. We followed the official workflow and loaded the normalized count from Seurat to CellChat for processing.

### 4.18 Western Blot

Total cellular proteins were extracted after lysing the cells for 30 min with RIPA lysis solution (AP01L014, Life‐iLab, China) and PMSF (AP02L0140, Life‐iLab). Use BCA assay kit to determine the total protein concentration according to the instructions. All protein samples were boiled for 10 min after adding the loading buffer (AP14L136, Life‐iLab) for denaturation. After treatment, the proteins were separated on YoungPAGE (M00938, GenScript) and then transferred to a PVDF membrane. The membranes were blocked with experimental quick‐sealing fluid (AP36L108, Life‐iLab) for 30 min. Antibodies were diluted in normal antibody diluent (WB500D, New Cell & Molecular Biotech, China). The primary antibody was incubated overnight at 4°C, and the secondary antibody was incubated for 1 h at room temperature. The blots were visualized with ECL Detection Reagent (SB‐WB004, Share‐bio, Shang Hai).

Detailed antibody information is as follows: anti‐Col3a1 (R23957), anti‐FSP1 (342551), anti‐P53 (345567), anti‐LC3B (R381544), anti‐APG5 (R23497), anti‐RIP (R25595), anti‐MLKL (R380559), anti‐NLRP3 (R381207), anti‐GSDMD (R40136), anti‐GPX4 (R381958), anti‐FTH1 (R23306), anti‐PTGS2 (R23971), anti‐β‐actin (R380624), anti‐CD9 (R380441), anti‐HSP70 (R24633), anti‐TSG101 (R25999), anti‐Calnexin (340144), anti‐H3 (R24572), anti‐HMGB1 (R380710), anti‐HSP110 (R381496), anti‐HSP60 (201060), anti‐DLST (670492) were purchased from Zen‐bioscience (Chengdu, China). Anti‐GAPDH (abs124037) was from Absin. Anti‐Beclin‐1 (WL02508), anti‐Caspase1 (WL03450), anti‐TOMM20 (1:2000, WL03626), anti‐TNF‐a (WL01581), anti‐NF‐κB p65 (WL01980), anti‐P‐NFκB p65 (WL02169) were purchased from Wanleibio (Shenyang, China). Anti‐IL‐1α (DF6893) and anti‐Caspase3 (DF6879) were from Affinity Biosciences. Anti‐Col1a1 (1:500, HA722517), anti‐BAX (ET1603‐34), anti‐TGFβ (HA721143) were purchased from HUABIO (Hangzhou, China). Anti‐Smad2 (71269, GenuIN) and anti‐p‐Smad2 (61703, GenuIN) were from Genuine Biotechnologies. Anti‐IL‐1β (1:500, P012728) was purchased from Epizyme (Shanghai, China). Anti‐TLR4 (BYmab‐17807), anti‐MyD88 (BYmab‐13947), anti‐TRAF6 (BYmab‐04253) were purchased from BYabscience (Nanjing, China). All antibodies were used at a dilution of 1:1000 unless otherwise specified.

### 4.19 Statistical Analysis

All results were expressed as the mean ± standard error of the mean (SEM). Data were analyzed, and figures were produced with GraphPad Prism v9.5 (GraphPad Software, CA, USA). A two‐tailed unpaired Student t test or one‐way ANOVA was used to evaluate the data. *P *≤ 0.05 was considered significant (*P < 0.05, **P < 0.01, ***P < 0.001).

## Author Contributions

Jianfeng Ma, Mailin Gan, Shuang Liang, and Lijun Sun conceptualized this study; Jianfeng Ma, Mailin Gan, and Shuang Liang performed most of the experiments; Ziling Hao, Yiting Yang, Jianfeng Ma, and Mailin Gan performed data analysis and data visualization; Siyu Chen, Jiaxin Li, Qihang Wu, Yuqian Shi, and Lijun Sun performed part of the experiments. Jiaxin Li, Saihao Wang, and Yan Wang provided technical support. Xiaofeng Zhou, Lei Chen, and Ye Zhao provided experimental resources. Lijun Sun, Mailin Gan, and Li Zhu acquired funding and co‐supervised this study. Jianfeng Ma and Shuang Liang wrote the original manuscript with contributions from all authors. Li Zhu and Lijun Sun revised the manuscript. All the authors read and approved the final manuscript.

## Funding

This work was supported by National Key R&D Program of China (2024YFF1000201); Sichuan Science and Technology Program (2021ZDZX0008, 2021YFYZ0030, 2024NSFSC1176); China Agriculture Research System (CARS‐35); Pig Industry Technology System Innovation Team of Sichuan Province (SCCXTD‐2025‐8).

## Conflicts of Interest

The authors declare no conflicts of interest.

## Supporting information




**Supporting File 1**: advs74859‐sup‐0001‐SuppMat.docx.

**Supporting File 2**: advs74859‐sup‐0002‐Data.zip.

## Data Availability

The data that support the findings of this study are available from the corresponding author upon reasonable request.

## References

[1] S. V. Adams , S. M. Quraishi , M. M. Shafer , et al., “Dietary Cadmium Exposure and Risk of Breast, Endometrial, and Ovarian Cancer in the Women's Health Initiative,” Environmental Health Perspectives 122, no. 6 (2014): 594–600. 24633137 10.1289/ehp.1307054 PMC4050510

[2] K. Yu , S. Liu , Z. Lin , et al., “Effect of Trace Element Mixtures on the Outcome of Patients With Esophageal Squamous Cell Carcinoma: A Prospective Cohort Study in Fujian, China,” BMC Cancer 24, no. 1 (2024): 24. 38166697 10.1186/s12885-023-11763-9 PMC10762846

[3] S. Kumar and A. Sharma , “Cadmium Toxicity: Effects on Human Reproduction and Fertility,” Reviews on Environmental Health 34, no. 4 (2019): 327–338. 31129655 10.1515/reveh-2019-0016

[4] X. Gao , G. Li , X. Pan , et al., “Environmental and Occupational Exposure to Cadmium Associated With Male Reproductive Health Risk: A Systematic Review and Meta‐Analysis Based on Epidemiological Evidence,” Environmental Geochemistry and Health 45, no. 11 (2023): 7491–7517. 37584848 10.1007/s10653-023-01719-0

[5] W. Ali , Y. Ma , J. Zhu , H. Zou , and Z. Liu , “Mechanisms of Cadmium‐Induced Testicular Injury: A Risk to Male Fertility,” Cells 11, no. 22 (2022): 3601. 36429028 10.3390/cells11223601 PMC9688678

[6] J. K. Bhardwaj , A. Siwach , D. Sachdeva , and S. N. Sachdeva , “Revisiting Cadmium‐Induced Toxicity in the Male Reproductive System: An Update,” Archives of Toxicology 98, no. 11 (2024): 3619–3639. 39317800 10.1007/s00204-024-03871-7

[7] X.‐W. Li , S. Li , Y. Yang , et al., “The FAK/Occludin/ZO‐1 Complex Is Critical for Cadmium‐Induced Testicular Damage by Disruption of the Integrity of the Blood‐Testis Barrier in Chickens,” Journal of Hazardous Materials 470 (2024): 134126. 38554509 10.1016/j.jhazmat.2024.134126

[8] Y. Wang , J. Wu , M. Zhang , et al., “Cadmium Exposure During Puberty Damages Testicular Development and Spermatogenesis via Ferroptosis Caused by Intracellular Iron Overload and Oxidative Stress in Mice,” Environmental Pollution 325 (2023): 121434. 36907243 10.1016/j.envpol.2023.121434

[9] J. Zhou , L. Zeng , Y. Zhang , et al., “Cadmium Exposure Induces Pyroptosis in Testicular Tissue by Increasing Oxidative Stress and Activating the AIM2 Inflammasome Pathway,” Science of the Total Environment 847 (2022): 157500. 35870590 10.1016/j.scitotenv.2022.157500

[10] L. Zhou , H. Liu , Y. Chen , et al., “Unveiling Leydig Cell Heterogeneity and Its Role in Male Infertility: A Single‐Cell Transcriptomic Study of human Testicular Tissue,” Reproductive Biology 25, no. 1 (2025): 100972. 39566254 10.1016/j.repbio.2024.100972

[11] L. Zhang , M. Guo , Z. Liu , et al., “Single‐Cell RNA‐Seq Analysis of Testicular Somatic Cell Development in Pigs,” Journal of Genetics and Genomics 49, no. 11 (2022): 1016–1028. 35436608 10.1016/j.jgg.2022.03.014

[12] K. H. K. Choy , S. Y. Chan , W. Lam , et al., “The Repertoire of Testicular Extracellular Vesicle Cargoes and Their Involvement in Inter‐Compartmental Communication Associated With Spermatogenesis,” BMC Biology 20, no. 1 (2022): 78. 35351114 10.1186/s12915-022-01268-5 PMC8966158

[13] F. Aghajanpour , R. Soltani , A. Afshar , et al., “Sertoli Cell‐Conditioned Medium Can Improve Blood‐Testis‐Barrier Function and Spermatogenesis in Azoospermia Mice Induced by Scrotal Hyperthermia: An Experimental Study,” International Journal of Reproductive BioMedicine 22, no. 1 (2024): 17–30. 38544670 10.18502/ijrm.v22i1.15238 PMC10963876

[14] Y. Ma , Q.‐W. Ma , Y. Sun , and X.‐F. Chen , “The Emerging Role of Extracellular Vesicles in the Testis,” Human Reproduction 38, no. 3 (2023): 334–351. 36728671 10.1093/humrep/dead015

[15] S. S. Wright , P. Kumari , V. Fraile‐Ágreda , et al., “Transplantation of Gasdermin Pores by Extracellular Vesicles Propagates Pyroptosis to Bystander Cells,” Cell 188, no. 2 (2025): 280–291.e17. 39742811 10.1016/j.cell.2024.11.018 PMC12272064

[16] N. Abu and S. N. Nasir , “Extracellular Vesicles and DAMPs in Cancer: A Mini‐Review,” Frontiers in Immunology 12 (2021): 740548. 34721407 10.3389/fimmu.2021.740548 PMC8554306

[17] M. Wang , X.‐F. Wang , Y.‐M. Li , et al., “Cross‐Talk Between Autophagy and Apoptosis Regulates Testicular Injury/Recovery Induced by Cadmium via PI3K With mTOR‐Independent Pathway,” Cell Death & Disease 11, no. 1 (2020): 46. 31969557 10.1038/s41419-020-2246-1 PMC6976559

[18] V. G. S. Mouro , V. A. Siman , J. D. Silva , et al., “Cadmium‐Induced Testicular Toxicity in Mice: Subacute and Subchronic Route‐Dependent Effects,” Biological Trace Element Research 193, no. 2 (2020): 466–482. 31030385 10.1007/s12011-019-01731-5

[19] S. H. Yang , J. B. He , L. H. Yu , et al., “Protective Role of Curcumin in Cadmium‐Induced Testicular Injury in Mice by Attenuating Oxidative Stress via Nrf2/ARE Pathway,” Environmental Science and Pollution Research International 26, no. 33 (2019): 34575–34583. 31650475 10.1007/s11356-019-06587-9

[20] C. Han , Y. Zhu , Z. Yang , S. Fu , W. Zhang , and C. Liu , “Protective Effect of Polygonatum Sibiricum Against Cadmium‐Induced Testicular Injury in Mice Through Inhibiting Oxidative Stress and Mitochondria‐Mediated Apoptosis,” Journal of Ethnopharmacology 261 (2020): 113060. 32569717 10.1016/j.jep.2020.113060

[21] L. Yi , X.‐J. Shang , L. Lv , et al., “Cadmium‐Induced Apoptosis of Leydig Cells Is Mediated by Excessive Mitochondrial Fission and Inhibition of Mitophagy,” Cell Death & Disease 13, no. 11 (2022): 928. 36335091 10.1038/s41419-022-05364-w PMC9637113

[22] G.‐X. Zhou , H.‐L. Zhu , X.‐T. Shi , et al., “Autophagy in Sertoli Cell Protects Against Environmental Cadmium‐Induced Germ Cell Apoptosis in Mouse Testes,” Environmental Pollution 270 (2021): 116241. 33321432 10.1016/j.envpol.2020.116241

[23] M. Wang , C. Q. Zhu , L. Zeng , et al., “Melatonin Regulates the Cross‐Talk Between Autophagy and Apoptosis by SIRT3 in Testicular Leydig Cells,” Biochemical and Biophysical Research Communications 555 (2021): 182–189. 33823364 10.1016/j.bbrc.2021.03.138

[24] D. Cai , J. Li , Z. Peng , et al., “Interplay of Ferroptosis, Cuproptosis, Autophagy and Pyroptosis in Male Infertility: Molecular Crossroads and Therapeutic Opportunities,” International Journal of Molecular Sciences 26, no. 8 (2025): 3496. 40331931 10.3390/ijms26083496 PMC12026609

[25] L. Zeng , J. Zhou , X. Wang , Y. Zhang , M. Wang , and P. Su , “Cadmium Attenuates Testosterone Synthesis by Promoting Ferroptosis and Blocking Autophagosome‐Lysosome Fusion,” Free Radical Biology and Medicine 176 (2021): 176–188. 34610361 10.1016/j.freeradbiomed.2021.09.028

[26] K. Jiang , Z. Fu , P. Tsourkas , et al., “Single‐Cell Resolution Uncovers Neighboring Cell Subtypes that Share Steroidogenic Capacity During Fetal Testis Development,” Proceedings of the National Academy of Sciences USA 122, no. 23 (2025): 2501392122. 10.1073/pnas.2501392122 PMC12167995 40460128

[27] M. Gan , J. Ma , Y. Lei , et al., “Single‐Cell RNA Sequencing and PANDORA‐Seq Reveal the Pivotal Role of piRNA in Heat Stress‐Induced Testicular Injury,” International Journal of Biological Macromolecules 319, no. Pt 4 (2025): 145674. 40609925 10.1016/j.ijbiomac.2025.145674

[28] J. A. Makela and R. M. Hobbs , “Molecular Regulation of Spermatogonial Stem Cell Renewal and Differentiation,” Reproduction 158, no. 5 (2019): R169–R187. 31247585 10.1530/REP-18-0476

[29] L. Wang , X. Li , T. Bu , et al., “Cadmium‐Induced Sertoli Cell Injury Through p38‐MAPK and Related Signaling Proteins—A Study by RNA Sequencing,” Endocrinology 164, no. 6 (2023): bqad045. 36928142 10.1210/endocr/bqad045

[30] S. H. Yang , S. T. Chen , C. Liang , et al., “Effects of Cadmium Exposure on Leydig Cells and Blood Vessels in Mouse Testis,” International Journal of Environmental Research and Public Health 19, no. 4 (2022): 2416. 35206604 10.3390/ijerph19042416 PMC8878469

[31] J. Zhou , Y. Zhang , L. Zeng , X. Wang , W. Xiang , and P. Su , “Cadmium Exposure Induces Pyroptosis of TM4 Cells Through Oxidative Stress Damage and Inflammasome Activation,” Ecotoxicology and Environmental Safety 270 (2024): 115930. 38184979 10.1016/j.ecoenv.2024.115930

[32] Y. Hai , J. Hou , Y. Liu , et al., “The Roles and Regulation of Sertoli Cells in Fate Determinations of Spermatogonial Stem Cells and Spermatogenesis,” Seminars in Cell & Developmental Biology 29 (2014): 66–75. 24718316 10.1016/j.semcdb.2014.04.007

[33] W. Ali , Y. Bian , H. Ali , et al., “Cadmium‐Induced Impairment of Spermatozoa Development by Reducing Exosomal‐MVBs Secretion: A Novel Pathway,” Aging 15, no. 10 (2023): 4096–4107. 37220720 10.18632/aging.204675 PMC10258001

[34] B. Cappe , M. Vadi , E. Sack , et al., “Systematic Compositional Analysis of Exosomal Extracellular Vesicles Produced by Cells Undergoing Apoptosis, Necroptosis and Ferroptosis,” Journal of Extracellular Vesicles 12, no. 10 (2023): 12365. 37807017 10.1002/jev2.12365 PMC10560658

[35] S. Yoon , A. Kovalenko , K. Bogdanov , and D. Wallach , “MLKL, the Protein That Mediates Necroptosis, Also Regulates Endosomal Trafficking and Extracellular Vesicle Generation,” Immunity 47, no. 1 (2017): 51–65.e7. 28666573 10.1016/j.immuni.2017.06.001

[36] L. Schaefer , “Complexity of Danger: The Diverse Nature of Damage‐Associated Molecular Patterns,” Journal of Biological Chemistry 289, no. 51 (2014): 35237–35245. 25391648 10.1074/jbc.R114.619304 PMC4271212

[37] B. Dukay , B. Csoboz , and M. E. Toth , “Heat‐Shock Proteins in Neuroinflammation,” Frontiers in Pharmacology 10 (2019): 920. 31507418 10.3389/fphar.2019.00920 PMC6718606

[38] S. Mihm , “Danger‐Associated Molecular Patterns (DAMPs): Molecular Triggers for Sterile Inflammation in the Liver,” International Journal of Molecular Sciences 19, no. 10 (2018): 3104. 30309020 10.3390/ijms19103104 PMC6213769

[39] W. Liang , S. Sagar , R. Ravindran , et al., “Mitochondria Are Secreted in Extracellular Vesicles When Lysosomal Function Is Impaired,” Nature Communications 14, no. 1 (2023): 5031. 10.1038/s41467-023-40680-5 PMC10439183 37596294

[40] B. C. Hammerling , R. H. Najor , M. Q. Cortez , et al., “A Rab5 Endosomal Pathway Mediates Parkin‐Dependent Mitochondrial Clearance,” Nature Communications 8 (2017): 14050. 10.1038/ncomms14050 PMC5290275 28134239

[41] A. Picca , F. Guerra , R. Calvani , et al., “Mitochondrial‐Derived Vesicles: The Good, the Bad, and the Ugly,” International Journal of Molecular Sciences 24, no. 18 (2023): 13835. 37762138 10.3390/ijms241813835 PMC10531235

[42] Y. Gao , N. Mi , W. Wu , et al., “Transfer of Inflammatory Mitochondria via Extracellular Vesicles From M1 Macrophages Induces Ferroptosis of Pancreatic Beta Cells in Acute Pancreatitis,” Journal of Extracellular Vesicles 13, no. 2 (2024): 12410. 10.1002/jev2.12410 PMC10847061 38320981

[43] Y. Shi , P. Luo , W. Wang , et al., “M1 But Not M0 Extracellular Vesicles Induce Polarization of RAW264.7 Macrophages via the TLR4‐NFκB Pathway In Vitro,” Inflammation 43, no. 5 (2020): 1611–1619. 32323096 10.1007/s10753-020-01236-7 PMC7476919

[44] B. Saha , F. Momen‐Heravi , I. Furi , et al., “Extracellular Vesicles From Mice With Alcoholic Liver Disease Carry a Distinct Protein Cargo and Induce Macrophage Activation Through Heat Shock Protein 90,” Hepatology 67, no. 5 (2018): 1986–2000. 29251792 10.1002/hep.29732 PMC5906190

[45] S. Min , J. Y. Kim , H. M. Cho , et al., “Heat Shock Protein 60 Couples an Oxidative Stress‐Responsive p38/MK2 Signaling and NF‐κB Survival Machinery in Cancer Cells,” Redox Biology 51 (2022): 102293. 35316673 10.1016/j.redox.2022.102293 PMC8943299

[46] Y. Peng , L. Li , J. Shang , et al., “Macrophage Promotes Fibroblast Activation and Kidney Fibrosis by Assembling a Vitronectin‐enriched Microenvironment,” Theranostics 13, no. 11 (2023): 3897–3913. 37441594 10.7150/thno.85250 PMC10334827

[47] A. Moreno‐Lanceta , M. Medrano‐Bosch , Y. Fundora , et al., “RNF41 Orchestrates Macrophage‐Driven Fibrosis Resolution and Hepatic Regeneration,” Science Translational Medicine 15, no. 704 (2023): abq6225. 10.1126/scitranslmed.abq6225 PMC10712730 37437019

[48] J. Ran , S. Yin , R. Issa , et al., “Key Role of Macrophages in the Progression of Hepatic Fibrosis,” Hepatology Communications 9, no. 1 (2025): 0602. 10.1097/HC9.0000000000000602 PMC11637753 39670853

[49] W. Guo , C. Yang , J. Zou , et al., “Interleukin‐1β Polarization in M1 Macrophage Mediates Myocardial Fibrosis in Diabetes,” International Immunopharmacology 131 (2024): 111858. 38492336 10.1016/j.intimp.2024.111858 PMC11330059

[50] X.‐D. Zhang , J. Sun , X.‐M. Zheng , et al., “Plin4 Exacerbates Cadmium‐Decreased Testosterone Level via Inducing Ferroptosis in Testicular Leydig Cells,” Redox Biology 76 (2024): 103312. 39173539 10.1016/j.redox.2024.103312 PMC11387904

[51] J. Ma , M. Gan , S. Chen , et al., “Metabolome and Transcriptome Profiling Reveal tRNA‐Derived Small RNAs Regulated Glutathione Metabolism in Intrauterine Growth‐Restricted Pigs,” International Journal of Biological Macromolecules 293 (2025): 139167. 39732235 10.1016/j.ijbiomac.2024.139167

[52] S. Chen , “Ultrafast One‐Pass FASTQ Data Preprocessing, Quality Control, and Deduplication Using fastp,” Imeta 2, no. 2 (2023): 107. 10.1002/imt2.107 PMC10989850 38868435

[53] T. Stuart , A. Butler , P. Hoffman , et al., “Comprehensive Integration of Single‐Cell Data,” Cell 177, no. 7 (2019): 1888–1902. 31178118 10.1016/j.cell.2019.05.031 PMC6687398

[54] P. L. Germain , A. Lun , C. G. Meixide , et al., “Doublet Identification in Single‐Cell Sequencing Data Using scDblFinder,” F1000Research 10 (2021): 979. 35814628 10.12688/f1000research.73600.1 PMC9204188

[55] I. Korsunsky , N. Millard , J. Fan , et al., “Fast, Sensitive and Accurate Integration of Single‐Cell Data With Harmony,” Nature Methods 16, no. 12 (2019): 1289–1296. 31740819 10.1038/s41592-019-0619-0 PMC6884693

[56] M. Jung , D. Wells , J. Rusch , et al., “Unified Single‐Cell Analysis of Testis Gene Regulation and Pathology in Five Mouse Strains,” Elife 8 (2019): 43966. 10.7554/eLife.43966 PMC6615865 31237565

[57] F. Murat , N. Mbengue , S. B. Winge , et al., “The Molecular Evolution of Spermatogenesis Across Mammals,” Nature 613, no. 7943 (2023): 308–316. 36544022 10.1038/s41586-022-05547-7 PMC9834047

[58] S. Lukassen , E. Bosch , A. B. Ekici , et al., “Characterization of Germ Cell Differentiation in the Male Mouse Through Single‐Cell RNA Sequencing,” Scientific Reports 8, no. 1 (2018): 6521. 29695820 10.1038/s41598-018-24725-0 PMC5916943

[59] S. Jin , M. V. Plikus , and Q. Nie , “CellChat for Systematic Analysis of Cell–Cell Communication From Single‐Cell Transcriptomics,” Nature Protocols 20, no. 1 (2025): 180–219. 39289562 10.1038/s41596-024-01045-4

