# Supplementary material for: Sertoli Cell‐Derived Extracellular Vesicles Orchestrate Cadmium‐Induced Testicular Inflammation and Fibrosis
Source: Adv Sci (Weinh). 2026 Mar 25;13(30):e22278. doi: 10.1002/advs.202522278 (PMC13248802; doi:10.1002/advs.202522278)
Supplement: Supplementary file 1 — Supporting File 1: advs74859‐sup‐0001‐SuppMat.docx. [file ADVS-13-e22278-s002.docx]

Supporting Information

**Sertoli cell-derived extracellular vesicles drive fibrosis via macrophage activation in testicular injury**

*Jianfeng Ma^1,2,†^, Mailin Gan^1,2,†,*^, Shuang Liang^1,2,†^, Siyu* Chen^1^*^,2^, Ziling Hao^1,2^, Yiting Yang^1,2^, Jiawei Lu^1,2^, Qihang Wu^1,2^, Yuqian Shi^1,2^, Lijun Sun^1,2^, Jiaxin Li, Saihao Wang^1,2^, Yan Wang^1,2^, Xiaofeng Zhou^1,2^, Lei Chen^1,2^, Ye Zhao^1,2^, Li Zhu^1,2,*^ and Linyuan Shen^1,2,*^*

**
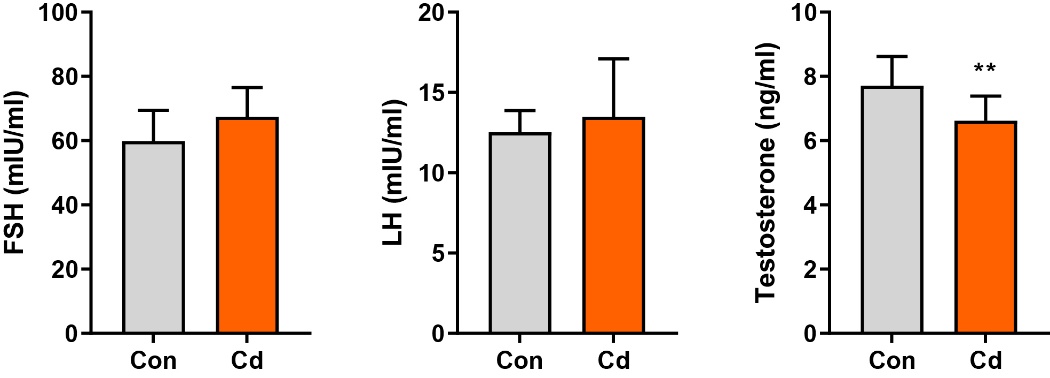
**

**Figure S1.** The levels of mice follicle stimulating hormone (FSH), luteinizing hormone (LH), and testosterone in plasma.


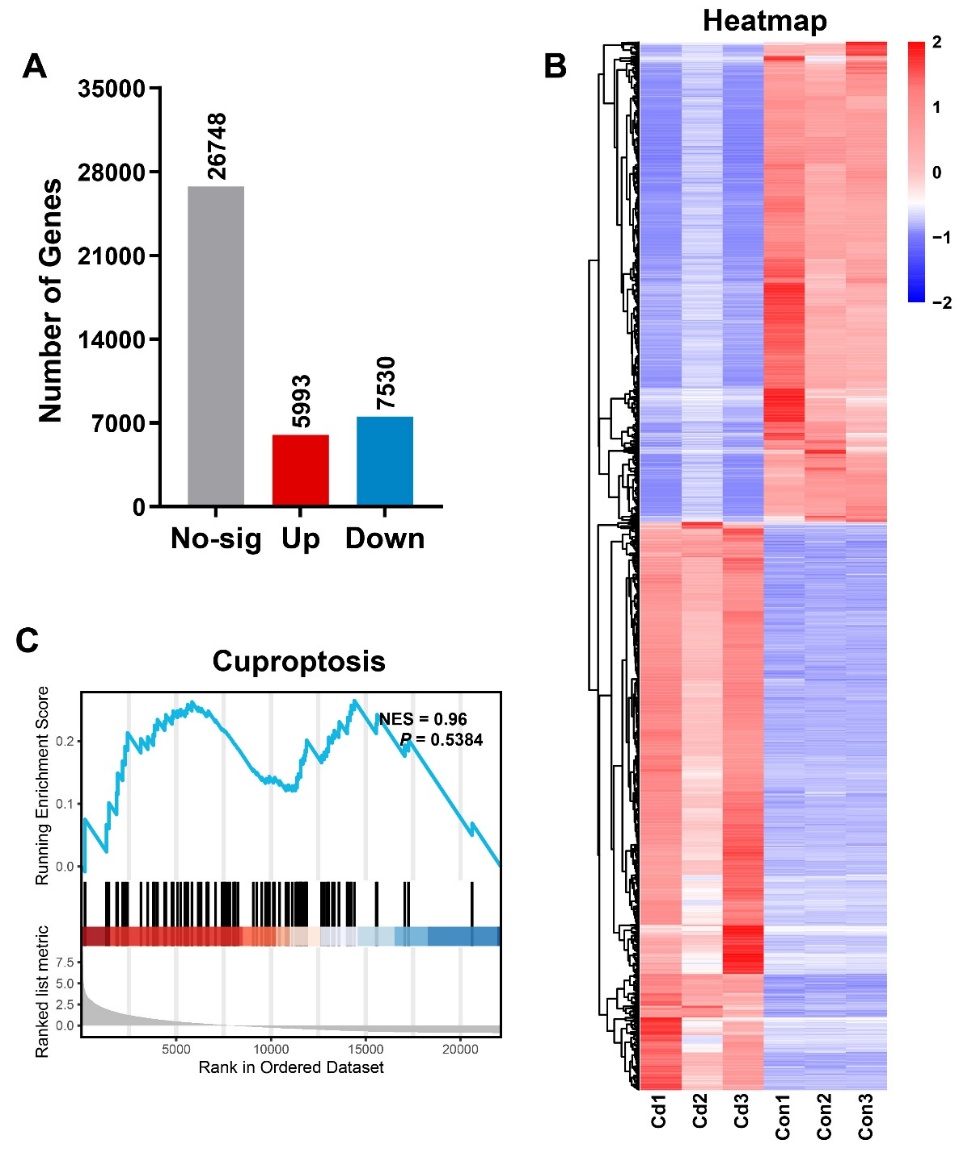


**Figure S2.** (A) Differential analysis of genes (DEGs) expression comparing Cd vs Con groups. (B) Heatmap of the DEGs. (C) Gene set enrichment analysis (GSEA) plot of cuproptosis.


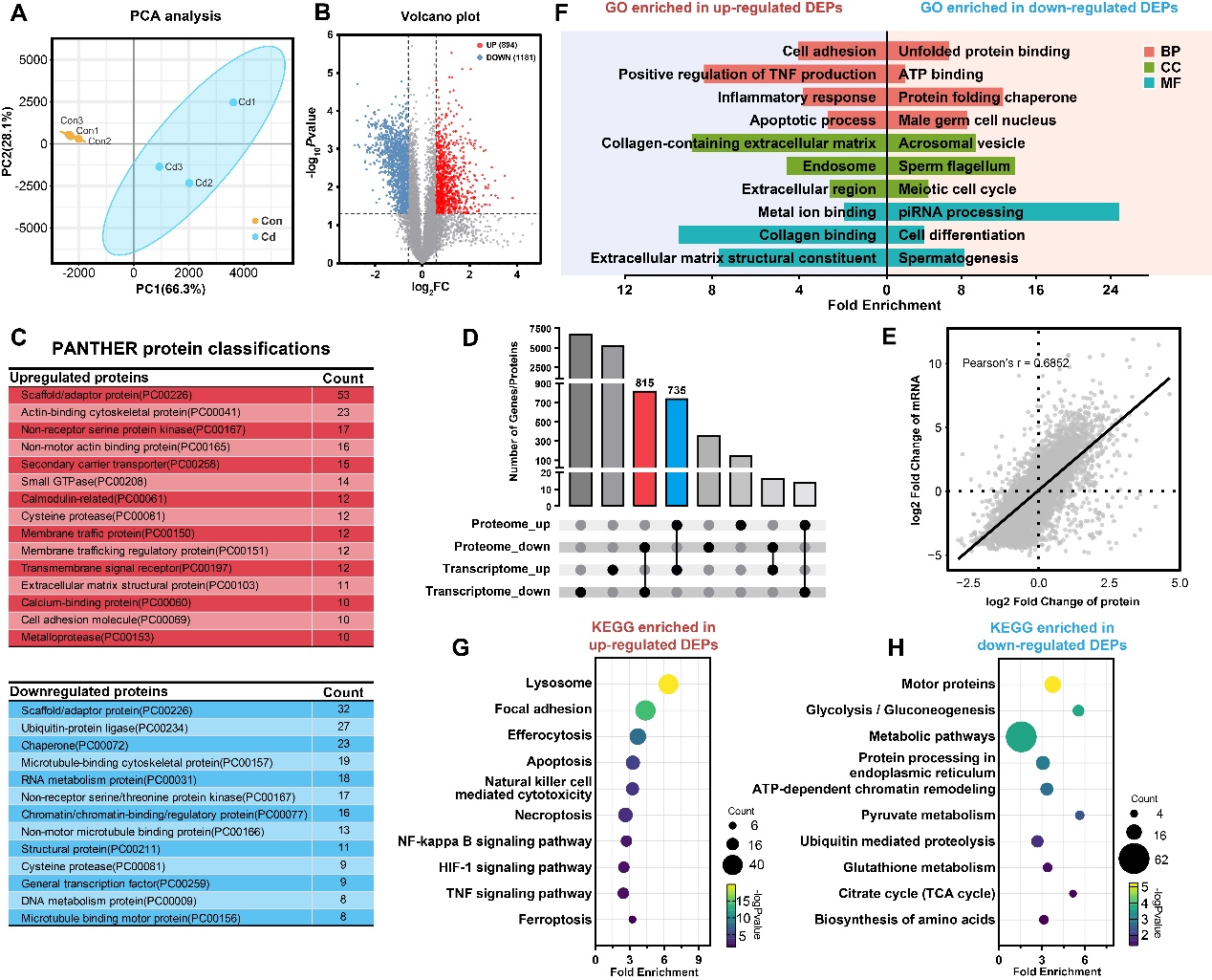


**Figure S3.** (A) Principal component analysis (PCA) score plots of proteome. (B) Volcano plot of the differentially expressed proteins (DEPs). (C) PANTHER protein class analysis of upregulated and downregulated proteins. (D) UpSet plot of the differentially expressed genes and proteins analysis. (E) Correlation analysis of the transcriptome and metabolome. (F) Gene Ontology (GO) enrichment analysis of the DEPs. KEGG enrichment analysis of upregulated (G) and downregulated (H) proteins.

**
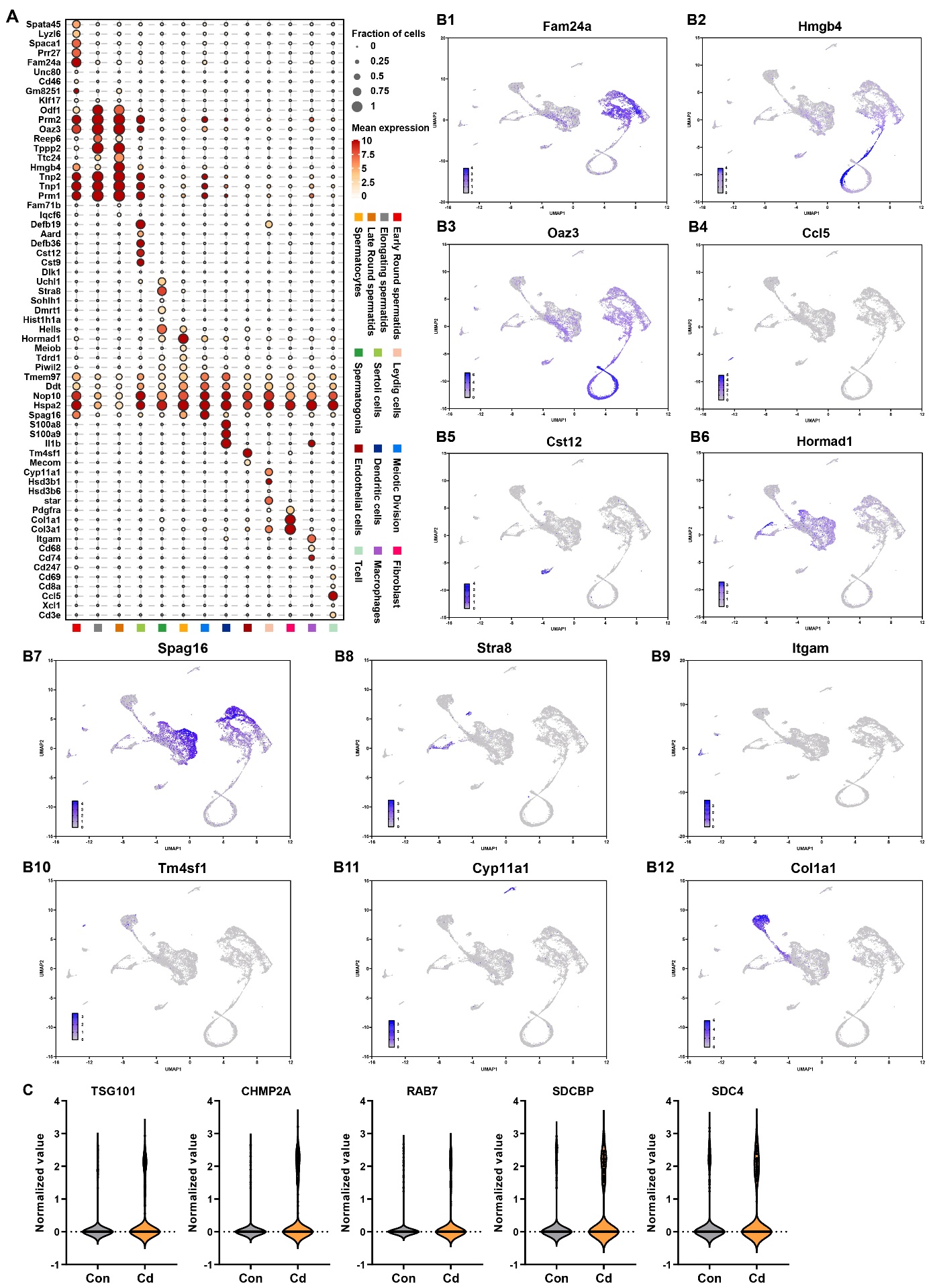
**

**Figure S4. (A)** Bubble plots of the marker genes expressed in the major cell types. The bubble size indicates the percentage of cells expressing a specific marker gene. The color represents the average non-integrated scaled gene expression within that cell population. **(B1-12)** UMAP plots showing expression of indicated genes. **(C)** Comparison of gene expression related to regulation of exosomal secretion in Sertoli cells.


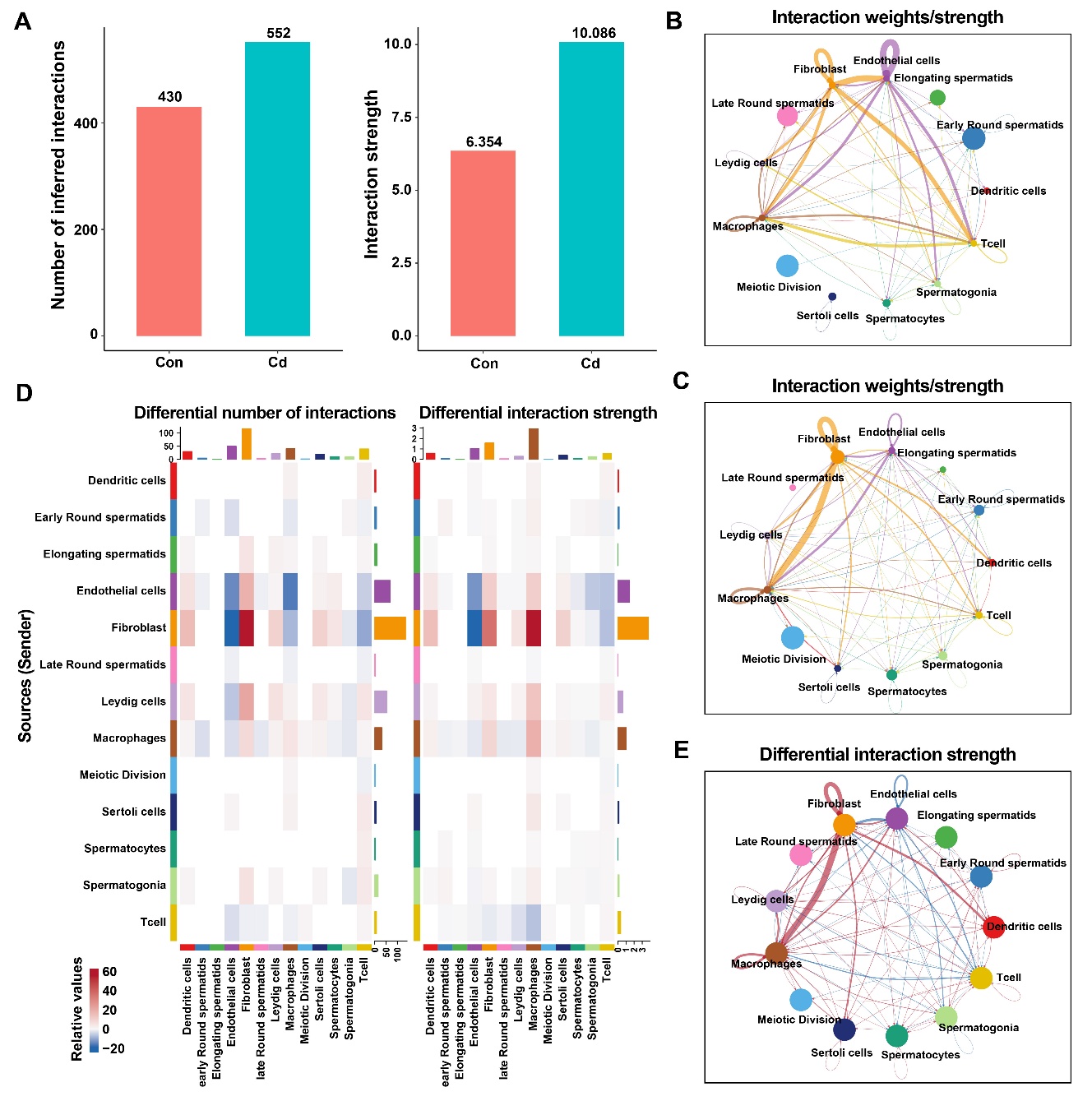


**Figure S5. (A)** The number of cell-to-cell interactions and the total interaction strength by CellChat software. Interaction network among major cell types constructed in control group **(B)** and Cd-exposed group **(C)** testis by CellChat. **(D)** The differential number of interactions and interaction strenth between the control group and Cd-exposed group testis. **(E)** The network of differential interaction strenth among major cell types.


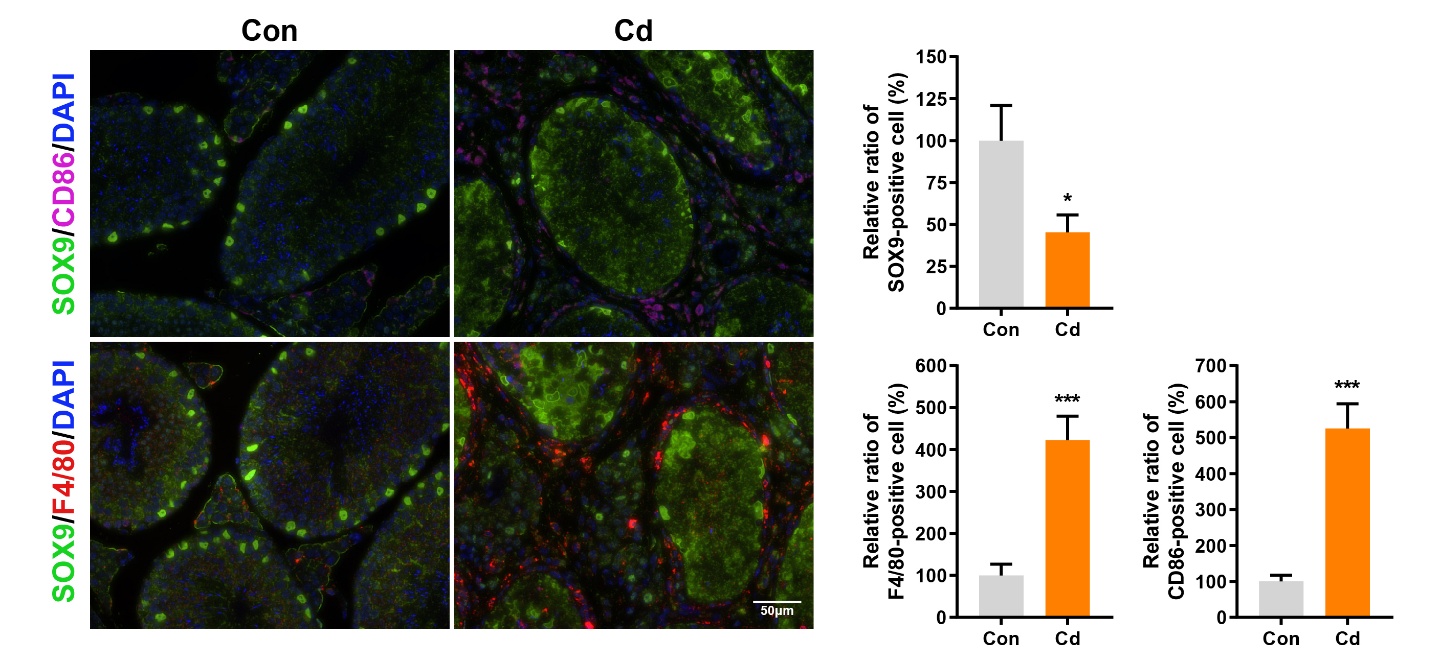


**Figure S6.** The immunofluorescent staining of F4/80 (red), SOX9 (green), and CD86 (purple) in the control and Cd exposure group, respectively. Results represent mean ± SEM, **p < 0.01, *p < 0.05, n=3.


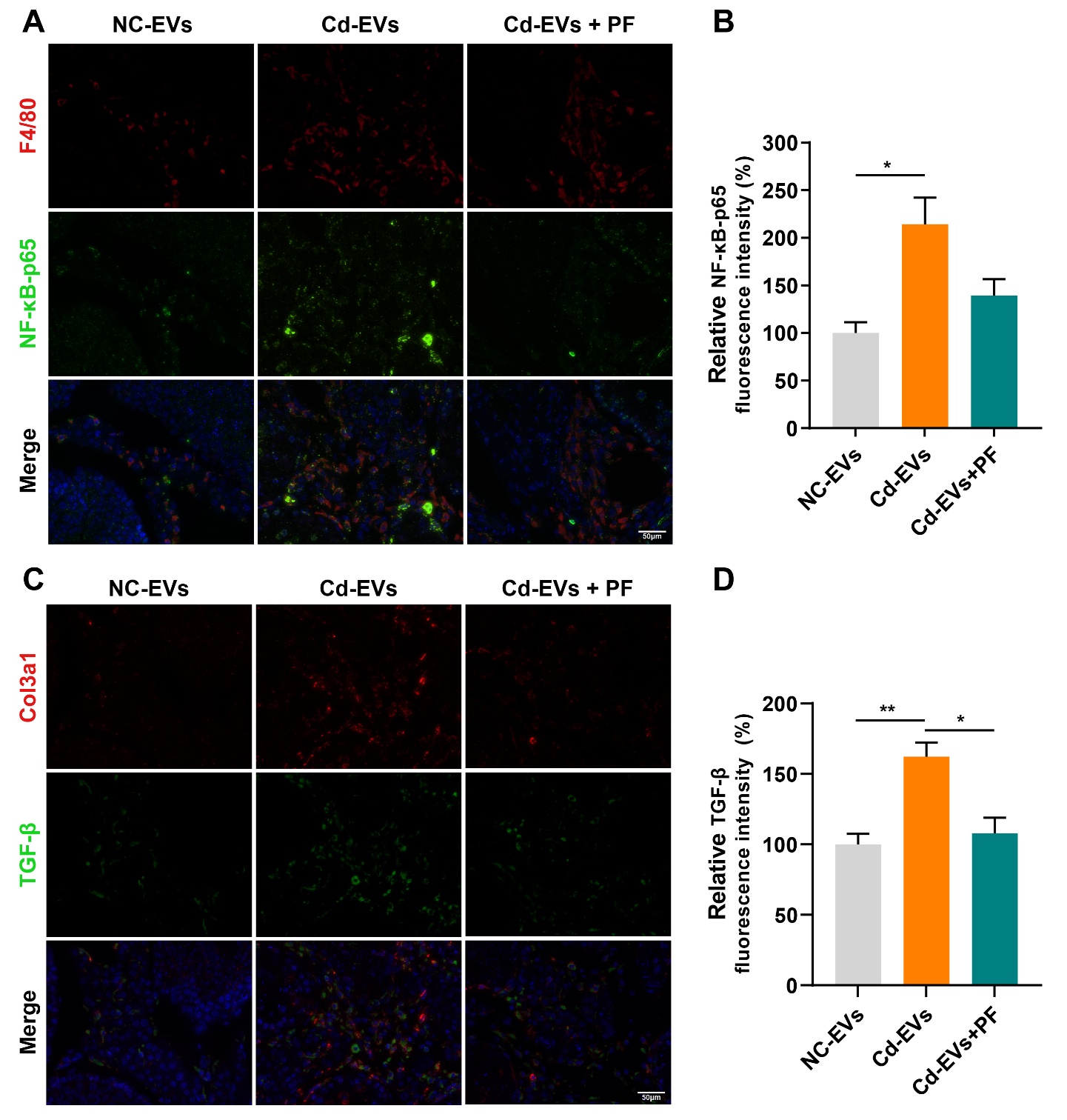


**Figure S7. (A)** The immunofluorescent staining of F4/80 (red) and NF-κB-p65 (green) in the testis. **(B)** Mean relative fluorescence intensity of NF-κB-p65 in F4/80-positive cells. **(C)** The immunofluorescent staining of Col3a1 (red) and TGF-β (green) in the testis. **(D)** Mean relative fluorescence intensity of TGF-β in Col3a1-positive cells. Negative control extracellular vesicles, NC-EVs. Cd-exposed Sertoli cell-derived extracellular vesicles, Cd-EVs. Cd-exposed Sertoli cell-derived extracellular vesicles and pirfenidone treatment, Cd-EVs+PF. Results represent mean ± SEM, **p < 0.01, *p < 0.05, n=3.


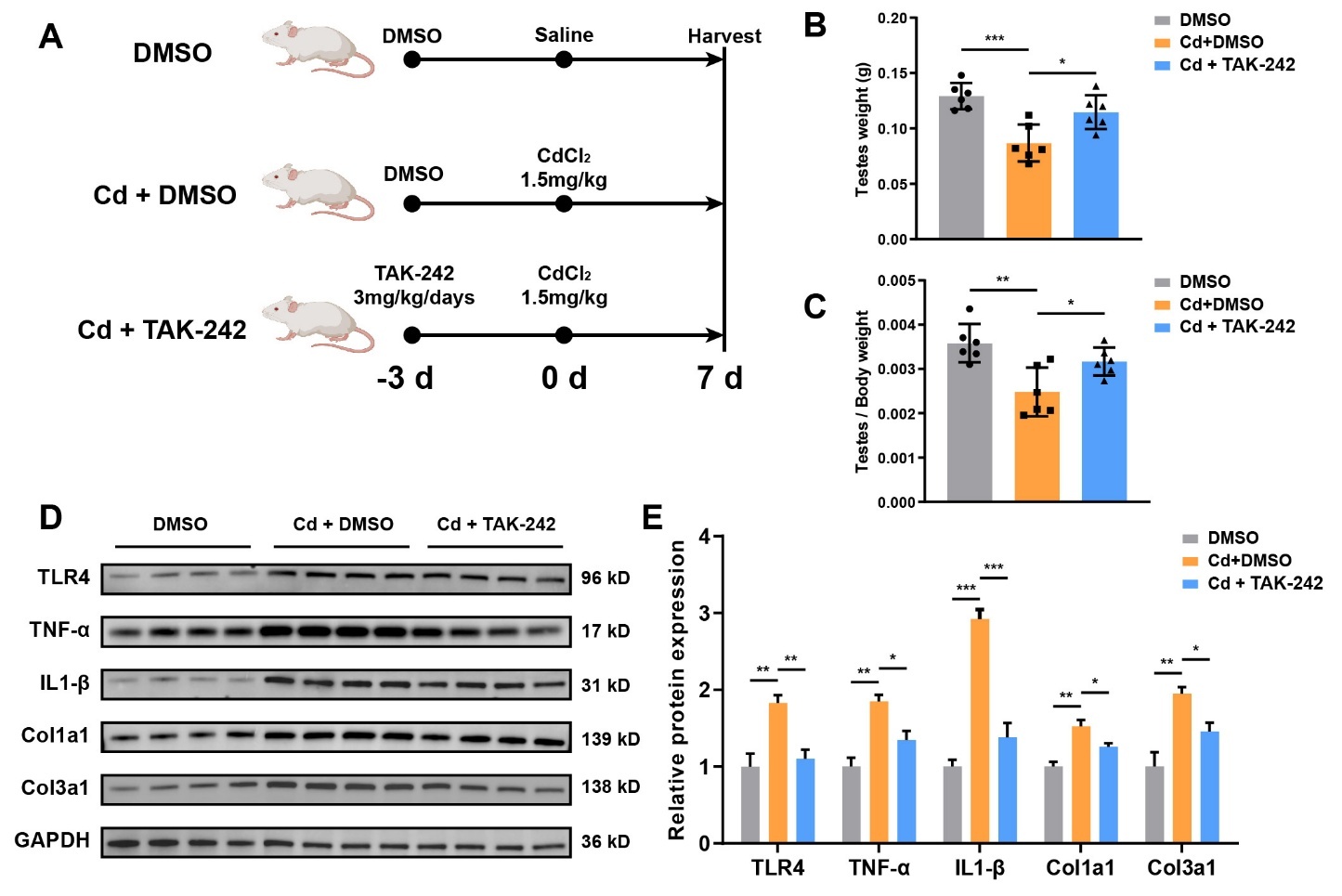


**Figure S8. (A)** A schematic diagram of the mice treatment administration process. **(B)** Comparison of testis weights. **(C)** Testis index in each group. **(D-E)** Western blot analysis and quantification for TLR4, TNF-α, IL-1β, Col1a1 and Col3a1 in the testis. Results represent mean ± SEM, ***p < 0.001, **p < 0.01, *p < 0.05, n=3.
