# Supplementary figures and images for: Sertoli Cell‐Derived Extracellular Vesicles Orchestrate Cadmium‐Induced Testicular Inflammation and Fibrosis
Source: Adv Sci (Weinh). 2026 Mar 25;13(30):e22278. doi: 10.1002/advs.202522278 (PMC13248802; doi:10.1002/advs.202522278)

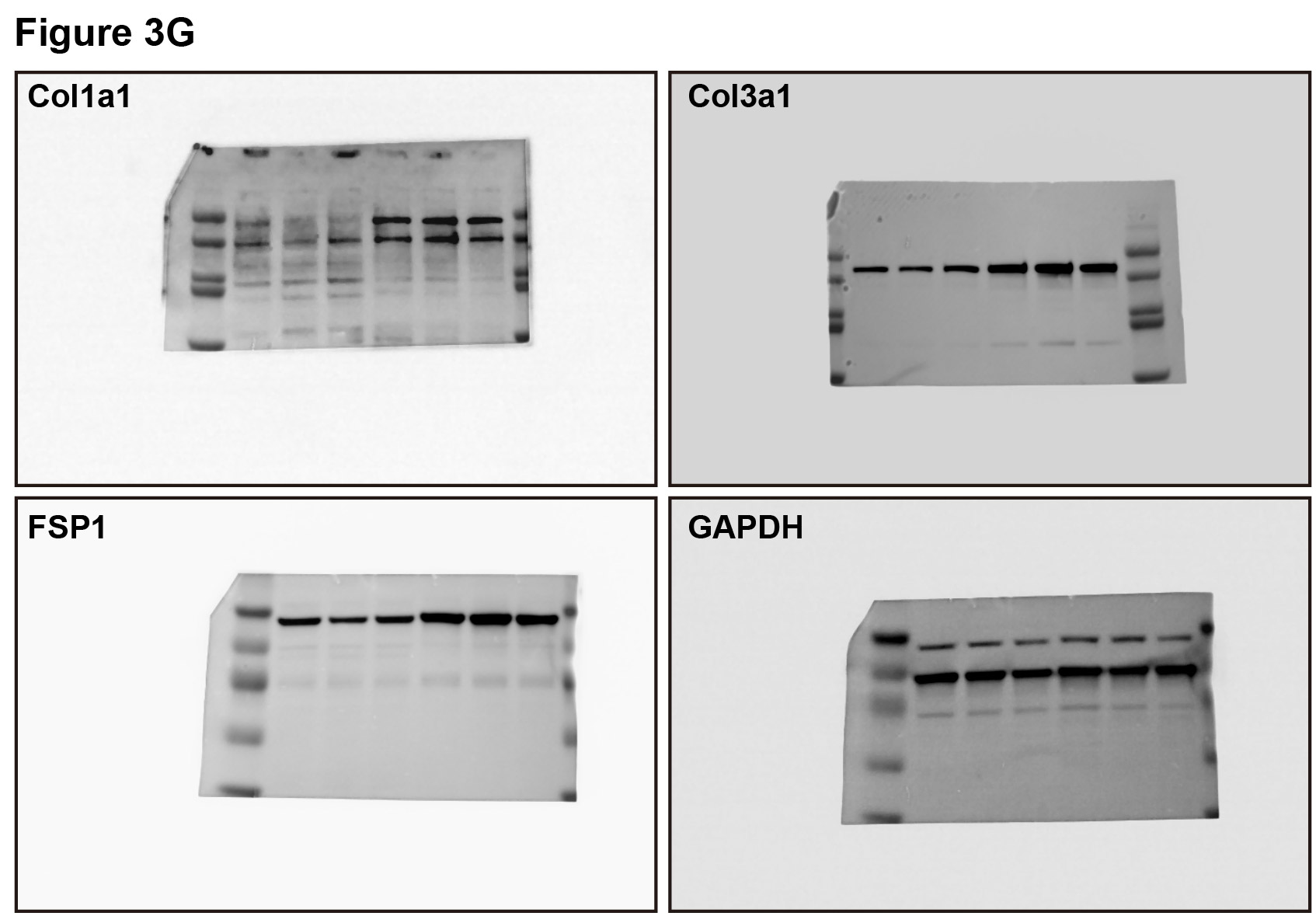


**
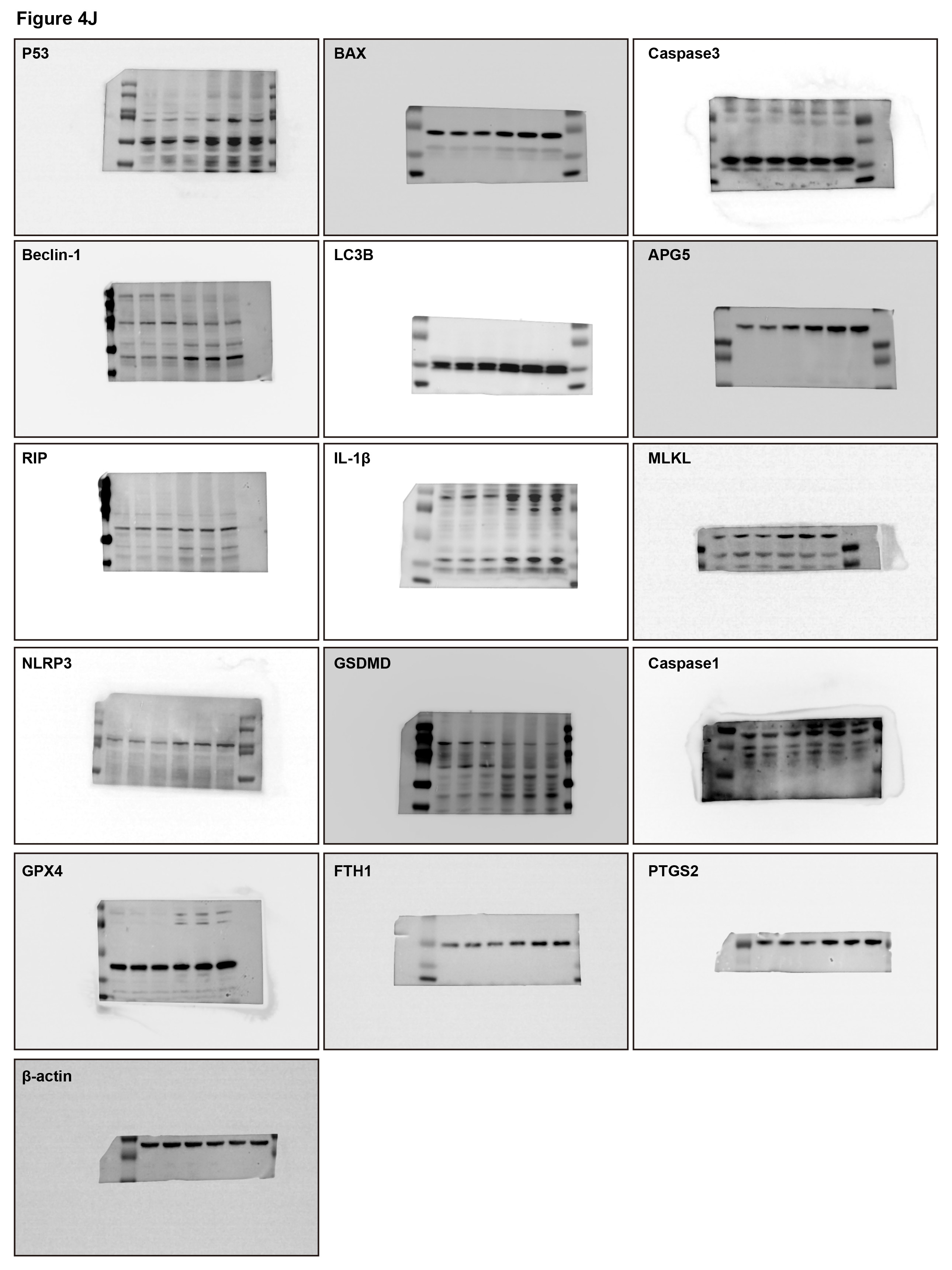
**

**
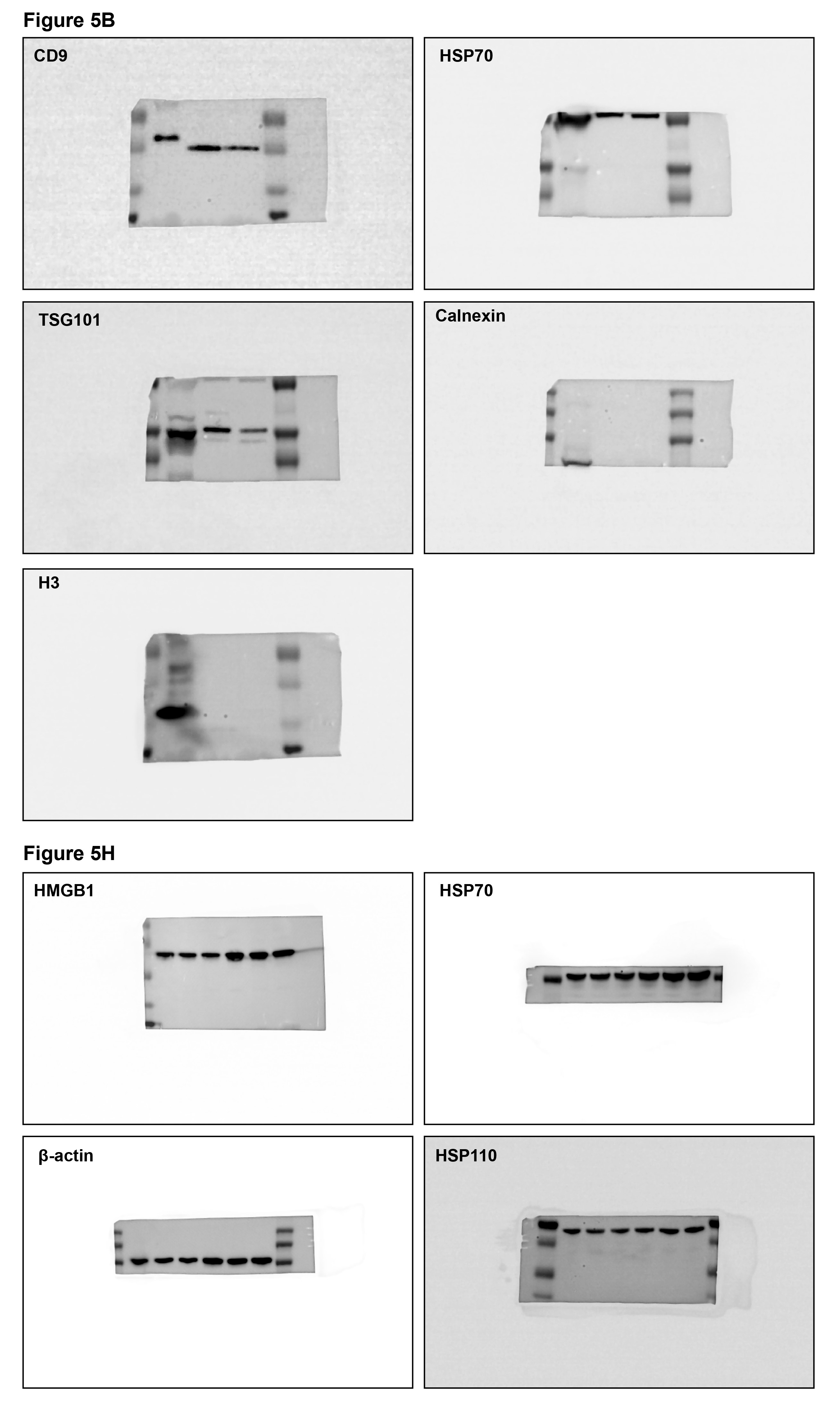
**

**
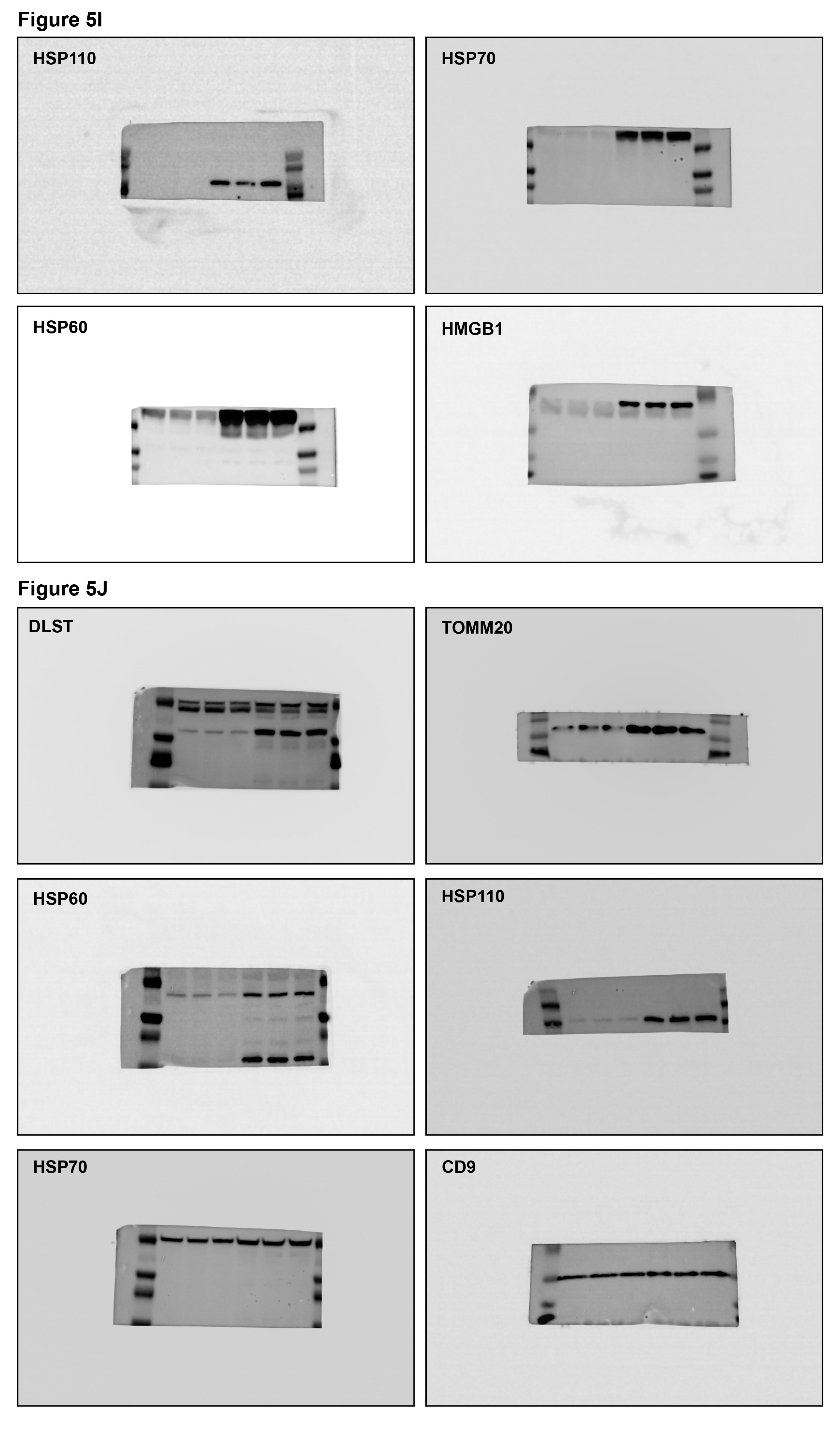
**

**
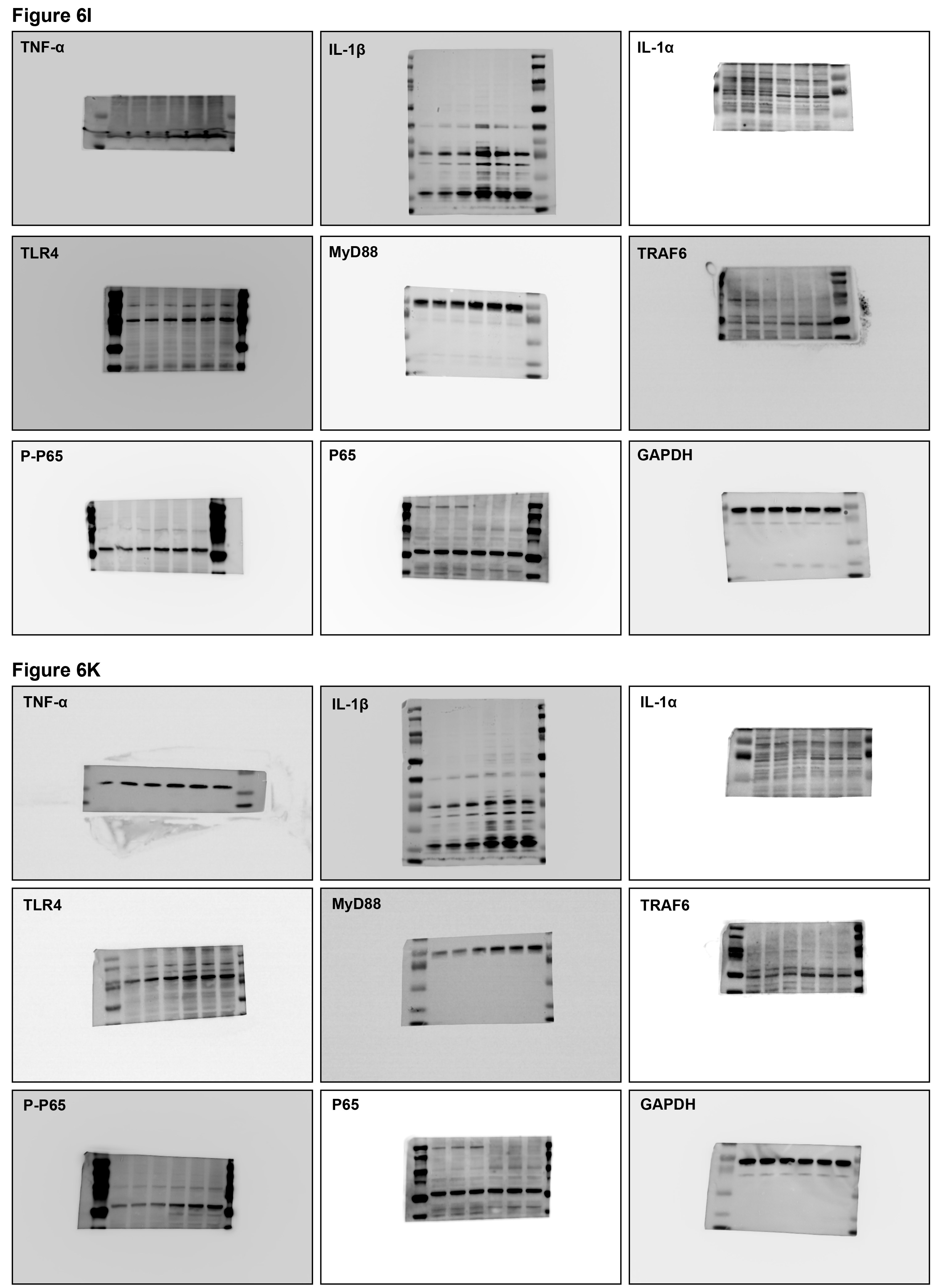
**

**
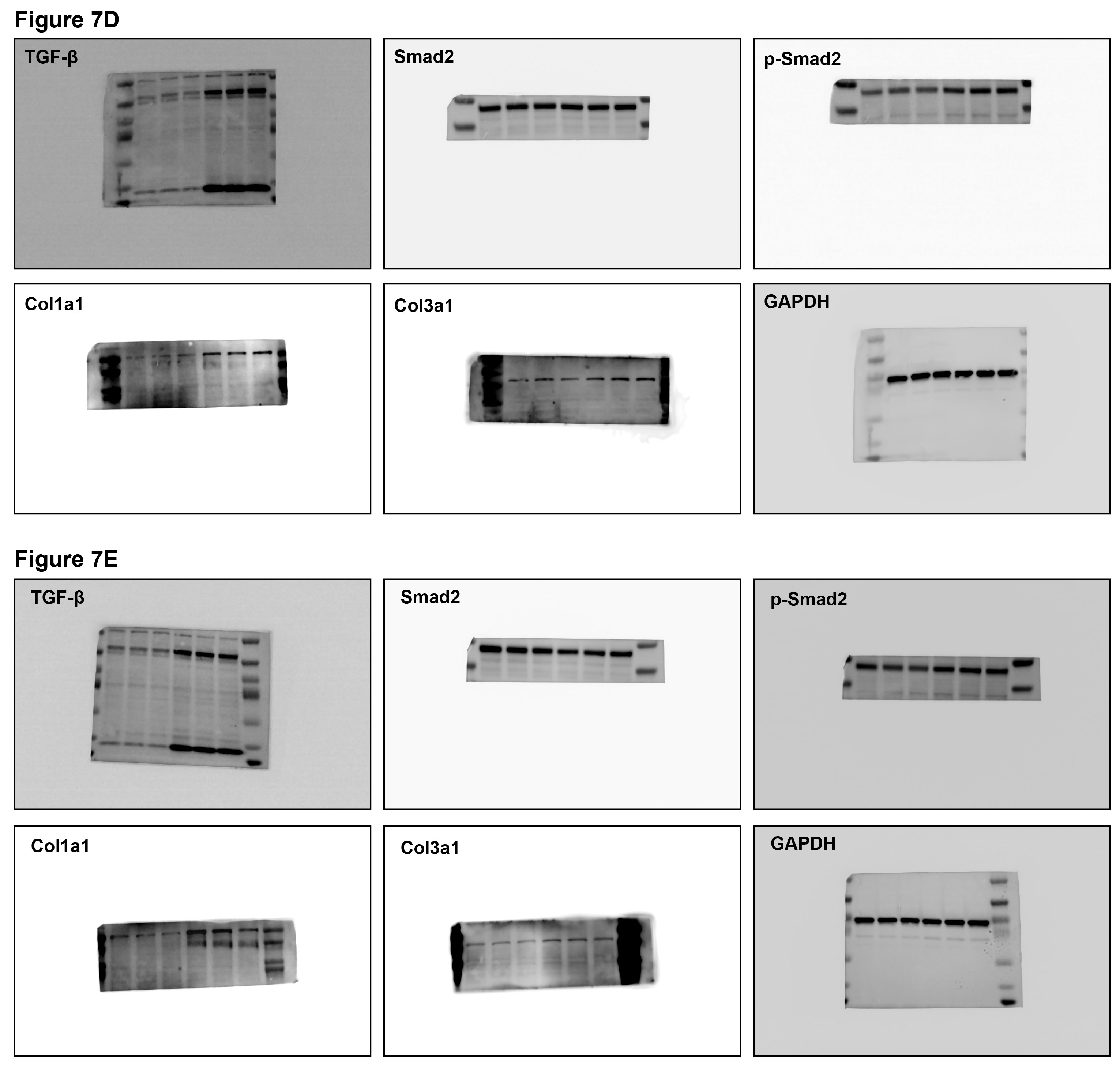
**

**
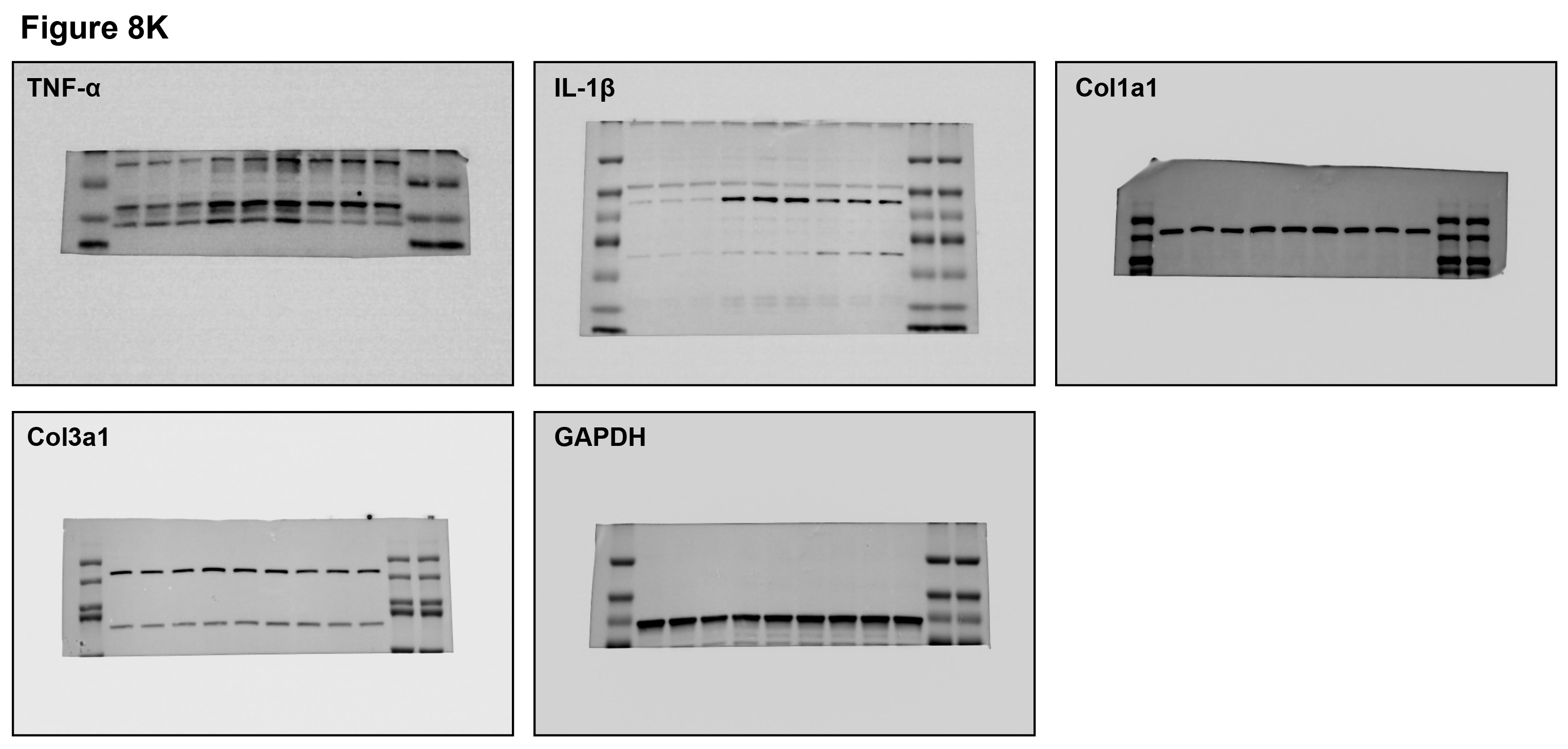
**

**
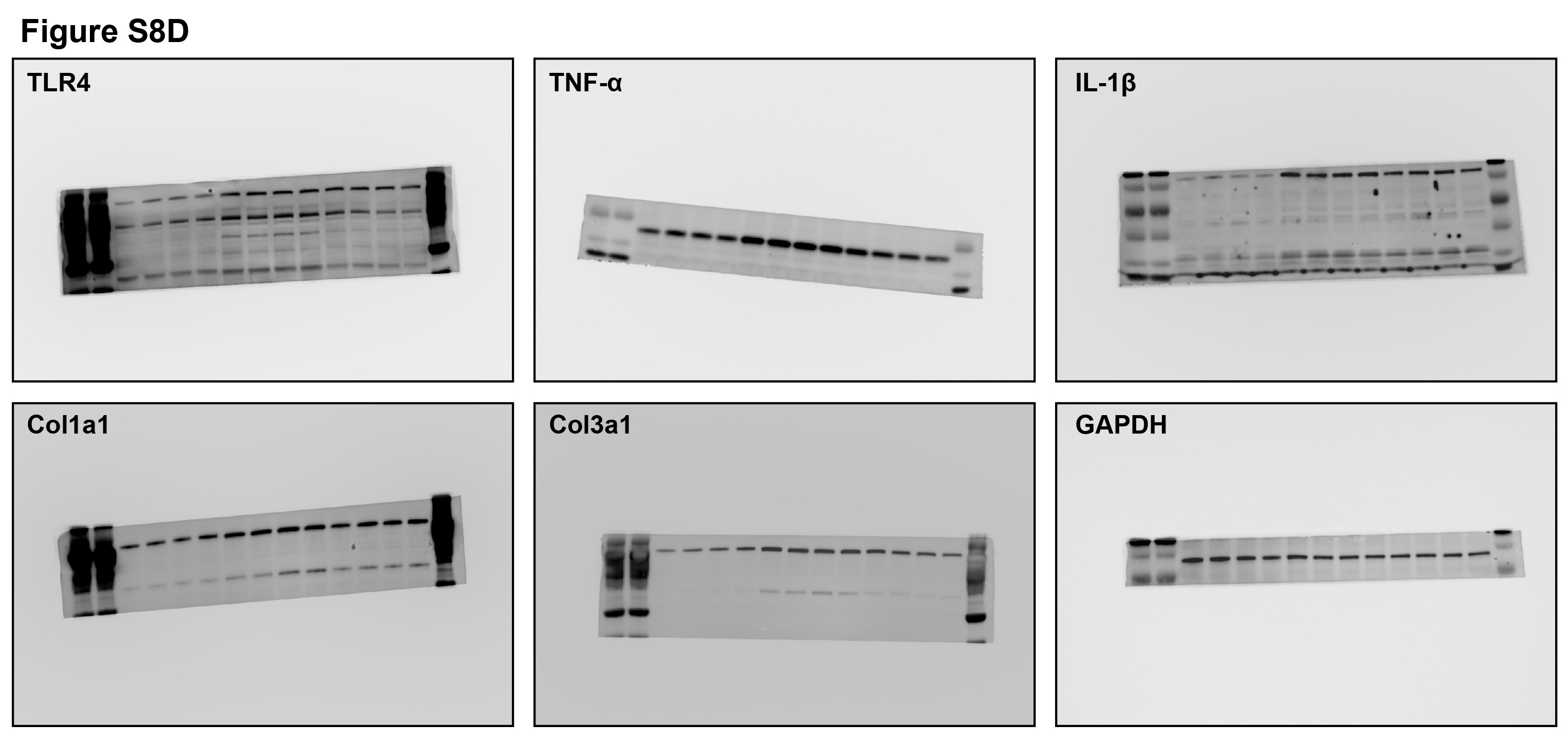
**

Supplement: Supplementary file 2 — Supporting File 2: advs74859‐sup‐0002‐Data.zip. [file ADVS-13-e22278-s001.zip › advs74859-sup-0002-Data/supplemental figure.docx]
